# Development of a Universal Platform for the Heterologous Expression of Bidirectional [Ni–Fe]-Hydrogenases in 

**DOI:** 10.1021/acssynbio.5c00150

**Published:** 2025-06-16

**Authors:** Dominik L. Siebert, Frank Sargent, Ammar Al-Shameri, Volker Sieber

**Affiliations:** † Chair of Chemistry of Biogenic Resources, TUM Campus Straubing for Biotechnology and Sustainability, Technical University of Munich, Schulgasse 16, 94315 Straubing, Germany; ‡ Microbes in Health and Disease, Biosciences Institute, Newcastle University, Framlington Place, NE2 4HH Newcastle upon Tyne, U.K.; § Catalytic Research Center, 9184 Technical University of Munich, Ernst-Otto-Fischer-Straße 1, 85748 Garching, Germany; ∥ SynBiofoundry@TUM, Technical University of Munich, Schulgasse 16, 94315 Straubing, Germany; ⊥ School of Chemistry and Molecular Biosciences, The University of Queensland, St. Lucia, Queensland 4072, Australia

**Keywords:** soluble hydrogenases, heterologous expression, enzyme maturation, Ni–Fe hydrogenase, artificial operon, H_2_ oxidation

## Abstract

Bidirectional [Ni–Fe]-hydrogenases are useful tools for integrating hydrogen into existing chemical processes by utilizing H_2_ to regenerate expensive cofactors such as NAD­(P)­H. One enzyme broadly applied to this purpose is the soluble [Ni–Fe]-hydrogenase from (*Cn*SH). However, the homologous production of *Cn*SH suffers from slow growth rates and complex growth medium requirements of the native host. In the present study, we developed a simple approach for the production of *Cn*SH in based on the coexpression of the maturation factors from . By optimizing the artificial operons coding for the hydrogenase proteins as well as the maturation factors, we were able to produce *Cn*SH with similar yields and activities compared to the native host. Additionally, we used our system to express three functional novel soluble [Ni–Fe]-hydrogenases, demonstrating its applicability for future enzyme screening and discovery.

## Graphical abstract

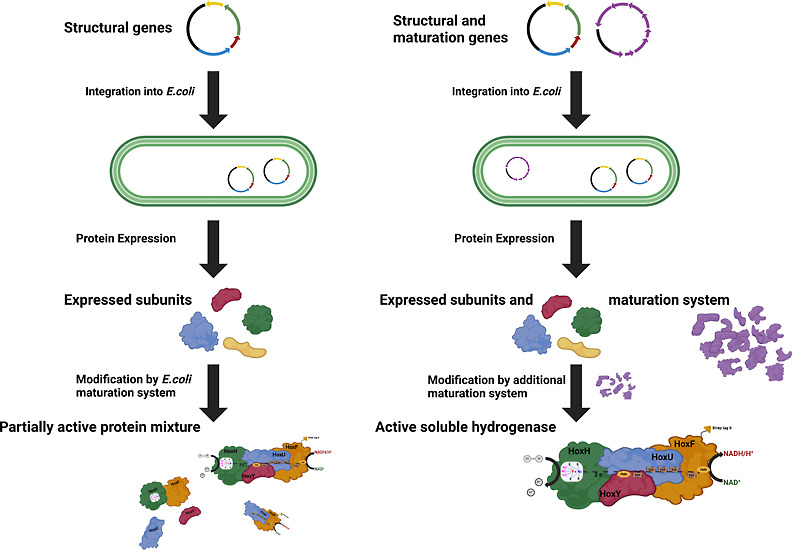

## Introduction

With increasing effects of climate change and an increased attention toward the carbon footprint of products and processes, the utilization of renewable resources is one of the grand challenges and essential for the development of sustainable chemistry. One of the key factors to this is the integration of green H_2_ into current processes. For biotechnological processes, such as whole cell and enzymatic catalytic processes, the integration of green H_2_ can be achieved by leveraging the potential of H_2_-converting enzymes, known as hydrogenases. Hydrogenases are classified by the type of metallic cofactor bound to their active site into three unrelated groups of [Fe]-,^1^ [FeFe]-,^2^ and [NiFe]-hydrogenases.^3^ While the majority of [FeFe]-hydrogenases are irreversibly deactivated in the presence of oxygen,^4,5^ some [NiFe]-hydrogenases have more tolerance towards oxygen.^6^ Bidirectional [NiFe]-hydrogenases are key tools used in biotechnological H_2_ applications, as they can shuttle the released electrons from H_2_ oxidation to various electron acceptors, including vital biological cofactors such as NAD­(P)^+^ and flavins.^7–9^ The soluble hydrogenase of (*CnSH*) is one of the most prominent [NiFe]-hydrogenases used for cofactor recycling,^10,11^ hydrogen production,^12,13^ and whole cell conversion.^14^ In its native form, *Cn*SH is a hexaheteromeric enzyme, consisting of the subunits HoxFUYHI_2_, which can be subdivided into three functional modules (Figure 1): The diaphorase module, consisting of HoxFU, converts NAD^+^ to NADH powered by the electron flow provided by the hydrogenase module (HoxYH), which is able to oxidize H_2_.^15^ The third functional subunit is a dimer of HoxI, which is suspected to have a regulatory function in the activation of the enzyme.^16^ While the preferred reaction of *Cn*SH is the H_2_-dependent reduction of NAD^+^, it is also known to perform the reverse reaction in the presence of NADH and the absence of hydrogen.^11^ To be functional, *Cn*SH requires a complex system of auxiliary proteins for building its metal cofactor, which consists of a set of maturation proteins HypA-F,^17–21^ an endopeptidase HoxW, and the auxiliary protein HypX,^22,23^ the latter being responsible for the aerobic maturation of the enzyme.^24^ Because of this complex maturation apparatus, the native remains the most common host for the expression of *CnSH* ^25–27^ and has also been used for the expression of other soluble hydrogenases.^28^ However, using for protein production is hampered by several limitations, including long cultivation time, complex molecular biology, and noncompatibility with standard growth media. Previously, a system for the heterologous expression of *CnSH* in was developed,^29^ which was based on the parallel expression of each subunit and auxiliary protein regulated by an individual set of T7 promoters and terminators. This resulted in high yields of an active *CnSH* but complicated further genetic modification or synergies with other genes, as each subunit would require an individual cloning step.

**Figure 1:**
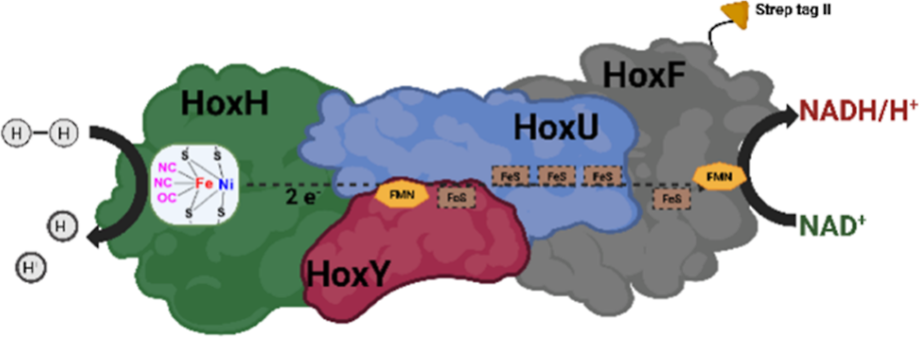
Schematic structure of *Cn*SH, its subunits, and its catalytic reaction.

In this work, we present the development of an operon-based genetic system for the functional production of *CnSH* in . Our aim of this system is to create a platform for the discovery and screening of other bidirectional [NiFe]-hydrogenases. As a starting point for our system, we used the constructs constructed by Lamont and Sargent (2017), which are based on the expression of an artificial operon under a common promoter created by linking the genes with artificial ribosome binding sites.^12^

## Results

The activity of the heterologously produced *Cn*SH depends on both the expression of the individual subunits HoxFUYHI as well as the presence and activity of the maturation proteins HypA-F, HoxW, and HypX in the cytoplasm of . Thus, we used two different types of expression systems. One system (pS) is a plasmid that carries the genes required for the expression of structural subunits under the control of the strong, inducible T7 promoter, and the second one (pM) carries the genes coding for the maturation proteins under the control of the weak, constitutive, *tatA* promoter. We optimized both plasmids individually, taking into consideration the yield and activity of the produced enzyme.

### Optimizing the Artificial Structural Operon

We were able to reproduce the data from the previously published system of Lamont and Sargent (2017); the expressed *Cn*SH in HJ002 cells showed activity within the cell-free lysate.^12^ Yet, the produced enzyme could not be purified by Ni-NTA affinity chromatography. Therefore, the His_6_-Tag linked to HoxF on the original plasmid (pS01) was substituted with a Strep-Tag to generate a new construct (pS02). Additionally, HoxN, an integral membrane Ni^2+^-transporter from ,^30^ was added to the original maturation plasmid pM01, containing the *Cn*SH maturation operon *hypA2* and HypX, generating pM02, since it was reported to have a positive effect on the activity of the regulatory hydrogenase from expressed in .^29,31^ Especially since the used strain BL21 (DE3) has mutations in the periplasmic binding-protein-dependent transport system for Ni^2+^ ions, decreasing their specific transport into the cytoplasm of the cells.^32,33^ Subsequently, BL21 (DE3) was transformed with pS02 and used for IPTG-induced protein expression. The hereby generated *Cn*SH could be purified using affinity chromatography with the Strep-tactin matrix.^11^ The SH showed activity on both NADH and NAD^+^ (Figure 2B,C); however, both the yields and activities were significantly lower than those reported in the literature for *Cn*SH.^32^ The yield of the SH is mainly dependent on the accessibility of the Strep-Tag connected to the HoxF subunit and the stability of the other subunits bound to HoxF. The oxidation of NADH is mainly dependent on the activity of the diaphorase HoxFU. However, the reduction of NAD^+^ is dependent on the amount of a fully assembled tetrameric enzyme and thereby is an indicator for a fully assembled, functional enzyme.

**Figure 2:**
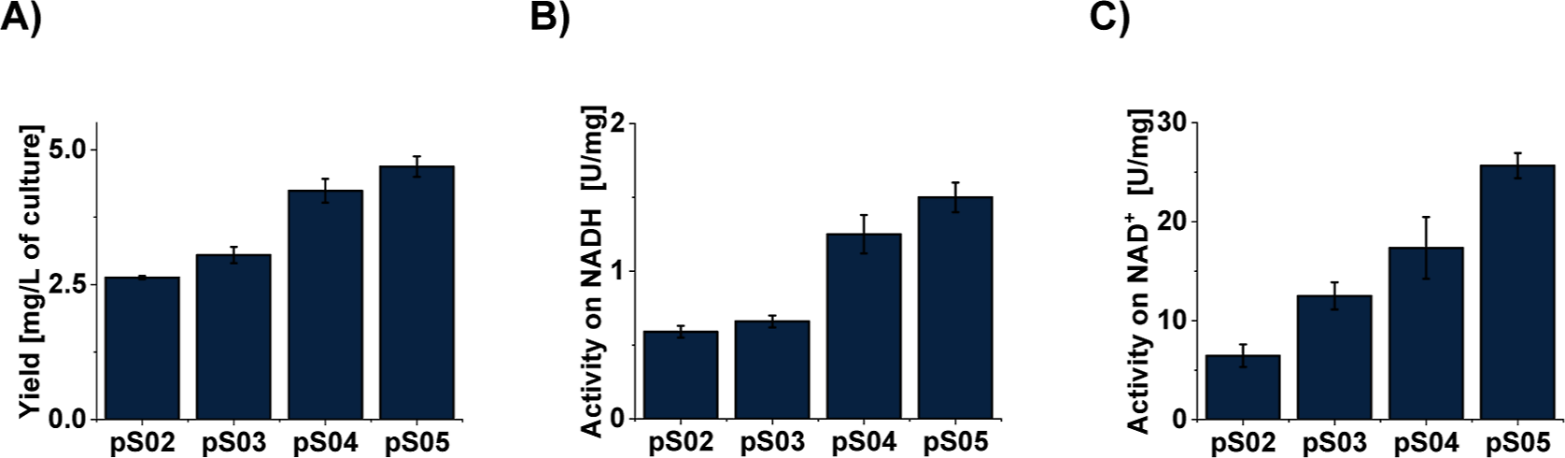
Effects of the design of the artificial expression operon on *Cn*SH yield and the activity of the produced enzyme. Showing the effect of different structural operons coexpressed with pM02 on the *Cn*SH yield (A), NADH oxidation activity (B), and hydrogen-driven NAD^+^ reduction activity (C).

We started by changing the backbone from pQE80 to pET22, generating pS03. This resulted in an increased enzyme yield and a higher activity of the produced enzyme. A possible reason for these changes could be the high stability of the pET22 plasmid compared to pQE80, as the pQE80 plasmid has shown a partial fragmentation, as shown in Figure S1. The next step was to shorten the linker between the Strep-Tag and HoxF, creating pS04. This further improved the yield and overall activities (Figure 2), which could indicate the shortened linker interfered less with the folding of the enzyme. Following this, we tested whether the removal of the gene coding for the HoxI subunit would influence the yield and activity of the enzyme, since the tetrameric enzyme HoxFUYH on its own is known to be active on NAD^+^/NADH.^16^ To test this, *hoxI* was removed from pS04, creating pS05. An expression of this plasmid resulted in a further increase in the yield and NAD^+^/NADH-dependent activities. Since the expression of pS05 has shown both the highest yield and the best activities of the produced enzyme, it was chosen for further experiments. All modified plasmids are listed in Table 1. The combination of pS05 and pM02 resulted in a specific activity comparable to that reported for native *Cn*SH and a yield exceeding 4 mg of the purified enzyme per liter of culture (Figure 2).

**Table 1:** Plasmids Used in This Work

plasmid	description	resistance	origin	genes	details
pM01	pSU-CN-HypA2X	kanamycin	P15A	*Cn*_HypA_2_B_2_F_2_C_1_D_1_E_1_X	Lamont and Sargent^12^
pM02	pSU-CN-HypA2XN	kanamycin	P15A	*Cn*_HypA_2_B_2_F_2_C_1_D_1_E_1_X HoxN	pM01 + HoxN
pM03	pSU-CN-HypA2XNW	kanamycin	P15A	*Cn*_HypA_2_B_2_F_2_C_1_D_1_E_1_X HoxNW	pM02 + HoxW
pM04	pSU-CN-HypA2XNW	kanamycin	P15A	*Cn*_HypA_2_B_2_F_2_C_1_D_1_E_1_X HoxN_CmW	pM02 + CmHoxW
pM05	pSU-CN-HypA2XNW	kanamycin	P15A	*Cn*_HypA_2_B_2_F_2_C_1_D_1_E_1_X HoxN_HpW	pM02 + HpHoxW
pS01	pQE80-His-AGGAGGA_8_—CNSH	ampicillin	ColE1	*Cn_*HoxFUYHWI	Lamont and Sargent^12^
pS02	pQE80-Strep-AGGAGGA_8_—CNSH	ampicillin	F1	*Cn_*HoxFUYHWI	pS01 → His_6_ changed to Strep
pS03	pET22-Strep-AGGAGGA_8_—CNSH	ampicillin	F1	*Cn_*HoxFUYHWI	pS02 → BB changed to pET22
pS04	pET22-Strep-AG -CNSH	ampicillin	F1	*Cn_*HoxFUYHWI	pS03→ linker shortened
pS05	pET22-Strep-AG-CN_HoxFUYHW	ampicillin	F1	*Cn_*HoxFUYHW	pS04 without HoxI
pS06	pET22-Strep-AG-CN_HoxFUYH	ampicillin	F1	*Cn_*HoxFUYH	pS05 without HoxW
pS07	pET22-Strep-AG-CM_HoxFUYH	ampicillin	F1	*Cm_*HoxFUYH	pS06 → with genes of *Cm*
pS08	pET22-Strep-AG-HP_HoxFUYH	ampicillin	F1	*Hp_*HoxFUYH	pS06 → with genes of *Hp*
pS09	pET22-Strep-AG-TX_HoxFUYH	ampicillin	F1	*Tx_*HoxFUYH	pS06 → with genes of *Tx*

Despite the fact that the produced *Cn*SH showed an activity of 25 U/mg, SDS-PAGE revealed an unequal distribution of the expressed subunits, especially HoxY. This could be an indicator of an uncompleted HoxH maturation as a result of a low expression level of the endopeptidase HoxW, as the *hoxW* gene is located on the furthest 3′ end of the operon. Putting *hoxW* on a constitutive prompter might solve this issue, as it could help guarantee a basal expression of the gene before the induction of the structural genes. To achieve this, the gene coding HoxW was transferred into pM02, resulting in the plasmid pM03, and shortening the plasmid pS05 resulted in the creation of pS06. Since the used maturation and structural plasmids are set apart by both copy number and expression levels, this resulted in a change in the intercellular HoxW concentration.

We compared the coexpression of the novel constructs to enzymes produced by pS05 coproduced with pM01 and pM02, respectively. Additionally, we tested the expression of pS05 without maturation plasmid as an additional negative control.

Interestingly, the negative control showed a slightly active *Cn*SH (Figure 3A). However, the coexpression of pM02 containing the *hypA2* operon and HoxN from increased the NAD^+^-dependent activity by a hundredfold and the yield by 5-fold compared to the negative control and showed a 50% improved activity compared to the cells lacking HoxN. This confirms the significance of both *hypA2* operon and HoxN from the native host for the activity of *Cn*SH in , despite that has its own set of Ni–Fe hydrogenases and the maturation machinery.^34–37^

**Figure 3:**
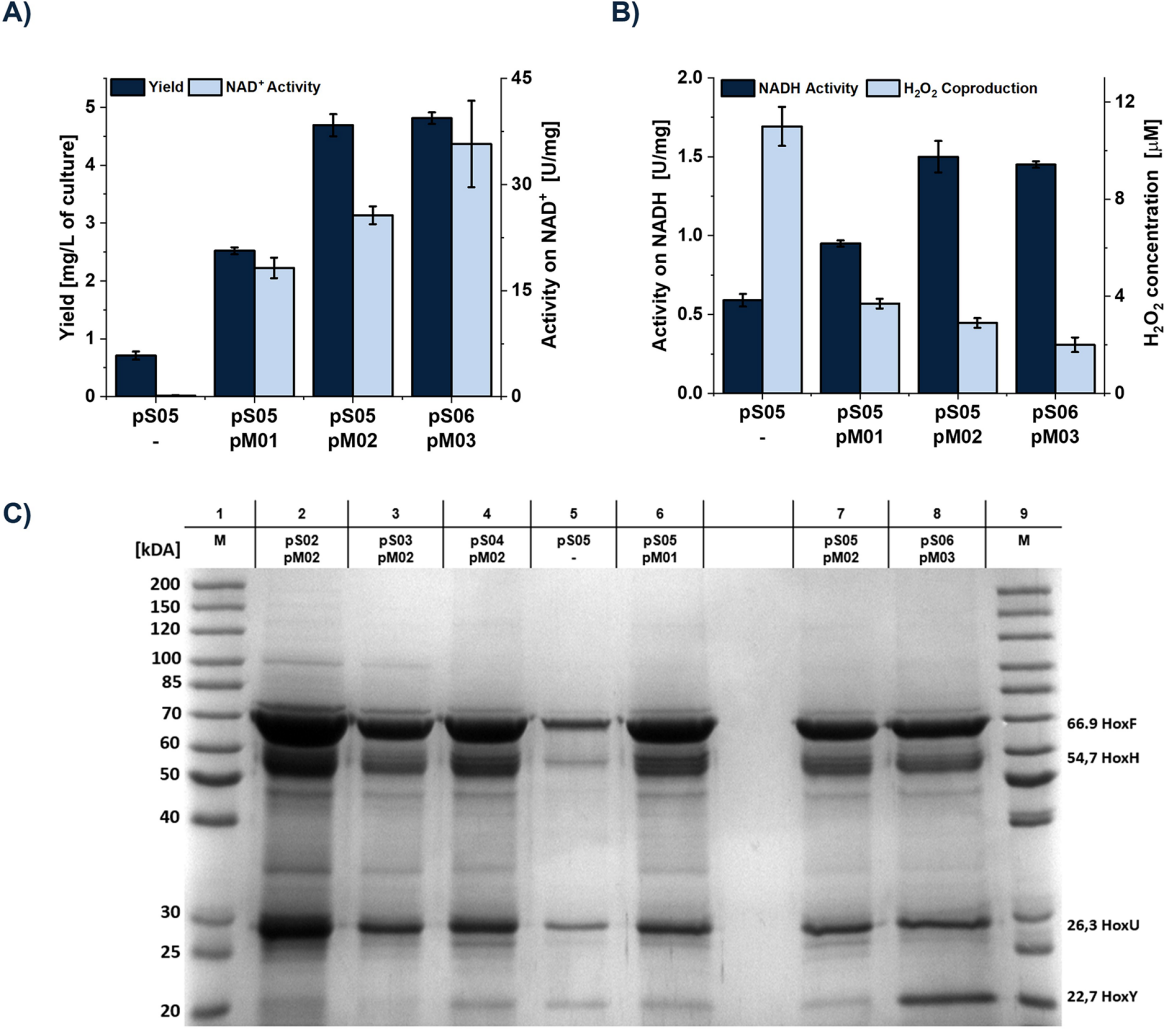
Comparison of *Cn*SH produced by the coexpression of the different maturational and structural operons. Produced enzymes are compared according to their yield, hydrogen-driven NAD^+^ reduction activity (A), activity on NADH oxidation with the coproduced amount of H_2_O_2_ per 1 mM oxidized NADH (B), and their purity and subunit composition via SDS-PAGE gel (C).

Furthermore, the coexpression of pM03 and pS06 provided a constitutive expression of *hoxW*, which led to a 40% increase in the hydrogen-driven NAD^+^ reduction activity (Figure 3A). In addition, it resulted in a significantly higher proportion of HoxY subunits (Figure 3C), resulting in a more balanced subunit distribution similar to *Cn*SH expressed in the native host.^100^

Furthermore, we analyzed the NADH oxidation activity and the H_2_O_2_ formation of the produced *Cn*SH. While there were close to no changes in the NADH oxidation activity, the amount of coproduced H_2_O_2_ was significantly less. This might indicate an improved reversed electron transfer toward hydrogen (Figure 3B) due to a fully assembled protein complex.^101^ By combining pM03 with pS06, we were able to increase the amount of *Cn*SH produced per liter of culture by 6.5-fold and the activity of the harvested enzyme by more than 200-fold compared to pS01 expressed without additional maturation factors.

### From *Cn*SH Production to a General SH Discovery System

After developing the genetic platform to produce active *Cn*SH in , we wondered whether it could be applied to identify and express further soluble bidirectional [NiFe]-hydrogenases. To find potential candidates, we searched in the databank for enzymes sharing a structural similarity with the four essential structural subunits (FUYH). We sorted the potential tetrameric enzymes by the averaged similarity across their subunits. This resulted in 206 potential bidirectional [NiFe]-hydrogenases containing an average similarity of at least 40%. A full list of all these candidates, including the UniProt entries of all their subunits and similarity comparison, is listed in (Table S5). Out of these candidates, we selected three enzymes to be expressed using our expression system.

First, we chose *Cm*SH from , a closely related enzyme with a similarity above 90% to *Cn*SH. Although it has a high similarity to *Cn*SH, *Cm*SH was until now mostly investigated in regard to its metabolic importance within its native host.^38^ Second, we expressed an unknown hydrogenase *Tx*SH from the thermostable betaproteobacterium .^39^ Last, we expressed *Hp*SH, a more distantly related enzyme, with a sequence similarity around 40% to *Cn*SH, from , an organism with a similar metabolism to *.* ^40^ The genes coding for the four essential structural proteins, HoxFUYH, of each of these enzymes were reverse translated from their primary amino acid sequence, codon optimized for , and integrated into a structural operon similar to pS06, resulting in pS07-pS09 for *Cm*SH, *Tx*SH, and *Hp*SH, respectively.

To test whether the resulting enzymes were actively expressed in our system, we analyzed the hydrogen-driven NAD^+^ reduction of the cell-free lysate created from coexpressing each of these cells with pM03 (Figure S4). To avoid false positive results induced by the native hydrogenases of , lysate expressing only pM03 was used as the negative control. All expressed novel hydrogenases showed a low but quantifiable activity in lysate, clearly differentiating from the background, indicating a successful expression. Interestingly, there was no observable correlation between the similarity to *Cn*SH and the measured activity, as both *Hp*SH and *Tx*SH have shown higher activities in cell-free lysate compared to *Cm*SH. As a next step, we tried to purify the novel hydrogenases using the procedure optimized for *Cn*SH, resulting in the protein samples shown in (Figure 4B) as well as the activities and yields with purified enzyme shown in (Figure 4A). Each of the novel hydrogenases could be purified with only minor impurities. Although the high concentration of HoxF compared with the other subunits implies a significant loss of enzyme integrity during purification. Furthermore, each of these enzymes could be produced in an active state, but both their production yields and activities on NAD^+^ were significantly lower compared to *Cn*SH. Out of these three enzymes, *Cm*SH showed the highest hydrogen-driven activity on NAD^+^ of around 0.8 U/mg. On the other hand, both *Tx*SH and *Hp*SH could be purified in higher amounts than *Cm*SH but showed close to no activity on NAD^+^ for *Hp*SH and *Tx*SH. Interestingly, their NADH oxidization activity reached above 0.8–1.6 U/mg similar to *CnSH*. The lack of activity of *Tx*SH on NAD^+^, despite that it contained all its four subunits, can hint toward a possible oxygen-induced inactivation of the active site. Therefore, we tried purifying *Tx*SH under anaerobic conditions. The purified enzyme showed a specific activity of 0.4 U/mg on NAD^+^ under standard conditions, which could be enhanced to up to 4 U/mg when the assay is performed at 45 °C (Figure 4A), the preferred temperature of its native organisms.^39^

**Figure 4:**
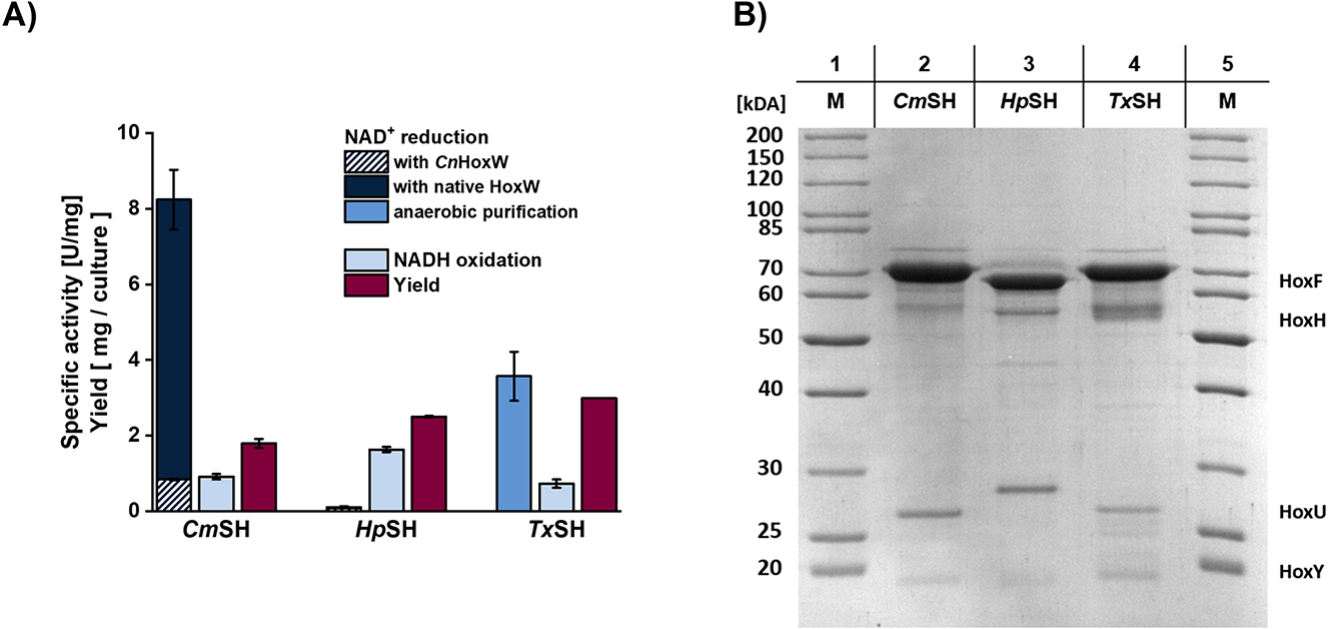
Comparison of the novel hydrogenases according to their yield and hydrogen-induced NAD^+^ reduction activity (A) and SDS-PAGE of the purified novel hydrogenases (B).

On the other hand, we hypothesized that HoxW most probably is strain/structure specific. This means that coexpressing *Hp*SH and *Cm*SH alongside their respective native HoxW should lead to better maturation and activity. For this, we integrated these endopeptidases into our maturational construct, creating pM4 and pM5, as described in Table 1. Following this, we expressed pS07 with pM4 and pS8 with pM5 and subsequently measured their respective activities on NAD^+^. For *Cm*SH, we observed a specific activity of around 8.2 U/mg, resulting in a 10-fold increase compared to the enzyme produced with *Cn*HoxW. The maturation of *Cm*HoxH was indeed improved (though still incomplete) by the coexpression of the native HoW (Figure S5), which contributed to the high activity. For *Hp*SH, an activity of around 0.1 U/mg could be measured, which is an increase of around 20% compared to the expression with *Cn*HoxW.

## Conclusions

In this work, we described the development of an expression system to produce active soluble hydrogenases. In contrast to existing systems,^29^ this system is not based on the parallel expression of individual subunits but rather on the expression of a whole artificial operon, comparable to other systems used for the expression of the regulatory hydrogenase of in *.* ^31,41–43^ Furthermore, our results confirm the beneficial effect of coexpressing HoxN for increasing the intracellular Ni^2+^ availability, resonating with previous studies.^29,31,41^ Considering the achieved specific activity of above 35 U/mg on NAD^+^ and yield of up to 5 mg/L, the amount and quality of *Cn*SH produced is within the range of the homologous production of the enzyme in *.* ^10^ However, we achieved a significantly higher space-time yield of *Cn*SH when considering the cultivation time required for .^44^ The artificial operon-based expression system facilitated the introduction of additional genes, the modification of individual sites, and the substitution of the whole set of structural genes, establishing the basis for the expression and screening of further soluble hydrogenases in . Three of these enzymes could be expressed actively in . *Tx*SH showed its highest activity at 45 °C when it was purified anaerobically. This goes hand in hand with reported data of soluble hydrogenases from thermophilic organisms like ,^45^ which exhibit high activities at high temperatures but loses 50% of its activity at 2% oxygen, and of ,^46^ which shows high activity at 80 °C but retains only 32% of its activity at 1% oxygen. By anaerobically purifying *Tx*SH with a residual activity of around 10% of *Cn*SH, it was possible to prove the applicability of our system for producing oxygen-sensitive soluble hydrogenases.

By coexpressing *Cm*SH with its native endopeptidase, HoxW were able to increase its activity more than 10-fold, proving the importance of respective endopeptidases for future SH expressions. For *Hp*SH, it could be observed that even a coexpression with its native HoxW did not significantly increase the activity, showing further potential for optimizations of both expression and purification conditions. Furthermore, the coexpression of additional corresponding maturation proteins, i.e., HypX, from the native organisms could be investigated, as it has proven to have major effects on the activity of [Ni–Fe]-hydrogenases.^24,47^

Our work is providing a universal expression platform for the production of active bidirectional [Ni–Fe]-hydrogenases in , enabling the discovery, screening, characterization, and optimization of these enzymes while eliminating the need to utilize their often complex-to-cultivate native hosts. Thereby, we strive to simplify future utilizations of [Ni–Fe]-hydrogenases, as they are sustainable tools for cofactor recycling of NAD­(P)^+^, NAD­(P)­H, and flavins.^48^ This makes them precious catalysts for integrating hydrogen into sustainable biotechnological approaches.

## Materials and Methods

### Plasmid Generation

pSU-A2-XN (pM02) was generated by integrating the synthetic HoxN gene from into pSU-A2-X. Likewise, pQE80-Strep-SH was generated by substituting the His_6_ tag from pQE80-SH. All genetic modifications were performed by using insertion PCR and isothermal recombination. A list of utilized primers is provided in the supplements. pM01 and pS01 are based on previous work from Lamont and Sargent;^12^ the other used plasmid were developed over the course of this work are in-depth described within the results.

### Enzyme Expression and Purification

All enzymes used in this study were produced by *BL21­(DE3)* cells containing the corresponding plasmids, as listed in Table 1. All used types of media were prepared according to their compositions given in the Supporting Information in Table S7. The protein expression was started by transforming BL21­(DE3) with both plasmids. These cells were plated on LB-agar as well as the appropriate antibiotics and incubated overnight at 37 °C. From this plate, a single clone was picked to prepare a preculture in LB media containing the same antibiotics. This preculture was incubated for 18 h at 37 °C. Afterward, it was used to inoculate a main culture of 1 L of TB media containing appropriate antibiotics as well as 100 μM of NiCl_2_ and FeCl_3_ in 5 L baffled glass flasks. The cells were incubated at 37 °C, and once an OD of 2 was surpassed, the culture was cooled down to 25 °C for 30 min. Subsequently, IPTG was added to a final concentration of 1 mM. After 18 h of additional incubation, the cells were harvested by centrifugation at 10.000*g* and frozen at −80 °C until further usage. The Strep-Tag-based purification was performed by use of either Strep-Tactin Superflow high-capacity resin or Strep-TactinXT 4Flow high-capacity resin according to their individual protocols as described by their manufacturer. The attempted His_6_-TAG purification of enzyme produced by pS01 was performed by use of a prepacked HisTrap FF column according to the manufacturer’s protocol. For the anaerobic purification of *Tx*SH, the cells were cultivated and harvested as described above, but the cell disruption was solely performed by adding lysozyme under a nitrogen atmosphere; further steps of the protein purifications were performed using Strep-TactinXT 4Flow high-capacity resin according to their individual protocols in an anaerobic glovebox under not more than 0.1% oxygen. Protein concentrations were determined by Bradford assay (ROTI®Quant) according to the manufacturer’s instructions.

### Enzyme Assays

To measure the H_2_-driven NAD^+^ reduction, ROTILABO glass cuvettes were filled with 2 mL of reaction buffer containing 50 mM Tris HCl, 1 mM NAD^+^, 1 mM TCEP, and 1 μM FMN at pH 8; subsequently, the cuvette was airtightly sealed and saturated with H_2_ for at least 15 min. Afterward, the reaction was started by adding purified enzyme via a gastight Hamilton syringe. The reaction was performed at 30 °C, followed by measuring the absorption at 340 and 365 nm. The NADH oxidation was performed in 96 well-plates, in which the used reaction buffer containing 50 mM Tris HCl, 1 mM NADH, and 100 μM FMN at pH 8 was incubated at 30 °C for 5 min until the reaction was started by adding the purified enzyme solution. The reaction was performed at 30 °C, followed by measuring the change in absorption at 340 and 365 Protein concentrations were determined by Bradford assay (ROTI®Quant) according to the manufacturer’s instructions. The H_2_O_2_ formation was measured spectrophotometrically at 498 nm using a coupled enzyamtic assay of 5 U mL^–1^ Horseradish peroxidase with 0.5 mM 4-aminoantipyrin and 2 mM vanillic acid.

## Supplementary Material



## Notes

Genetic maps, protein sequences, selected unprocessed data, and a list of identified potential [Ni–Fe]-hydrogenases (PDF)
