# Supplementary material for: Development of a Universal Platform for the Heterologous Expression of Bidirectional [Ni–Fe]-Hydrogenases in
Source: ACS Synth Biol. 2025 Jun 16;14(7):2710–7. doi: 10.1021/acssynbio.5c00150 (PMC12281615; doi:10.1021/acssynbio.5c00150)
Supplement: Supplementary file 1 [file sb5c00150_si_001.pdf]

Supplemental Information to:

Development of an universal platform for heterologous expression of bidirectional [Ni-Fe]-Hydrogenases in *E.coli*

Dominik L. Siebert[a], Frank Sergeant[b], Ammar Al-Shameri[a,*], Volker Sieber[a,c,d,e,*]

a) Chair of Chemistry of Biogenic Resources, TUM Campus Straubing for Biotechnology and Sustainability, Technical University of Munich, Schulgasse 16, 94315, Straubing, Germany

b) Microbes in Health and Disease, Biosciences Institute, Newcastle University, Framlington Place, Newcastle upon Tyne NE2 4HH, UK

c) Catalytic Research Center, Technical University of Munich, Ernst-Otto-Fischer-Straße 1, 85748, Garching, Germany

d) SynBiofoundry@TUM, Technical University of Munich, Schulgasse 16, 94315, Straubing, Germany

e) School of Chemistry and Molecular Biosciences, The University of Queensland, St. Lucia, QLD, 4072, Australia

* Corresponding authors

sieber@tum.de

a.al-shameri@tum.de

Table of content

Supplemental Information to: 1

Development of an universal platform for heterologous expression of bidirectional [Ni-Fe]-Hydrogenases in *E.coli* 1

Gels utilised for the evaluation of plasmid stability 4

Plasmid cards of used constructs 5

Measured yields, activities and quantified coproducts 7

List of potential soluble [NiFe] hydrogenases 9

Cell free lysate activities of screened soluble hydrogenases 21

Media compositions 22

Utilised primers 22

Sequence of additional enzymes 23

Purity of additional purification of the novel SH 236

List of tables and figures

Figure S1. Agarose gels prepared to investigate the plasmid stability of the used backbones 4

Figure S2. Plasmid cards of the constructs containing the structural genes used to produce bidirectional soluble [Ni-Fe]- hydrogenase 5

Figure S3. Plasmid cards of the constructs containing the maturational genes used to produce bidirectional soluble [Ni-Fe]- hydrogenase 6

Table S1. Yiels of the individual purifications of soluble hydrogenases 7

Table S2. NADH oxidising activity of the individual purifications of soluble hydrogenases 7

Table S3. Hydrogen induced NAD^+ reduction activity of the individual purifications of soluble hydrogenases 8

Table S4. Coproduced H_2O_2 of the individual purifications of soluble hydrogenases 8

Table S5. List of potential bidirectional [NiFe]-Hydrogenases listed by their relative similarity to *Cn*SH 9

Table S6. Hydrogen induced NAD^+ reduction activity of cell free lysate samples of soluble hydrogenases 21

Figure S4.Hydrogen induced NAD^+ conversion by cell free lysate containing potential soluble [Ni-Fe}-hydrogenases(A) and the calculated activities based on these graph(B). 21

Table S7. Composition of medias used in this study 22

Table S8. List of utilised primers 22

Figure S5. SDS-PAGE of the novel SHs after Coexpression with HoxW 2622

List of abbreviations

**NAD^+/H:**	**nicotinamide adenine dinucleotide (oxidized/ reduced form)**
**pS / pM**	**Structural plasmid / maturational plasmid**
**H_2O_2**	**hydrogen peroxide**
**SDS-PAGE:**	**sodium dodecyl sulphate polyacrylamide gel electrophoresis**
**PCR:**	**polymerase chain reaction**
Cn:	**Cupriavidus necator**
Cm:	**Cupriavidus metallidurans**
Hp:	**Hydrogenophaga pseudoflava**
Tx:	**Tepidicella xavieri**

Gels utilised for the evaluation of plasmid stability

To determine the stability of the plasmids used for this work, the individual constructs were firstly cloned in *E.coli* DH5α and purified via the Gene JET Plasmid miniprep Kit following the standard protocol, additionally an additional gel extraction was performed to avoid any contaminations or fragments caused during the plasmid generation. Secondly, 100 ng of this purified plasmid was again transformed in *E.coli* DH5α, grown firstly on an agarose plate with suitable antibiotics overnight, then transferred to preculture tubes filled with LB-media containing the same antibiotics. After 18 hours of incubation, the cells were harvested, and the plasmids were again purified. The resulting plasmid was subsequently linearized by a suitable restriction enzyme and analysed via an agarose gel. Since a second band there could be observed during the linearization of the pQE80 plasmid, a certain degree of antifragmentation was assumed. Since this did not occur when using the pET22 Backbone, it was utilised for the construction of further plasmids.

Used for Backbones:

pQE80

Expected Fragment size
Linearised: 10.5 kb
Double cut: 5.9 + 4.6 kb

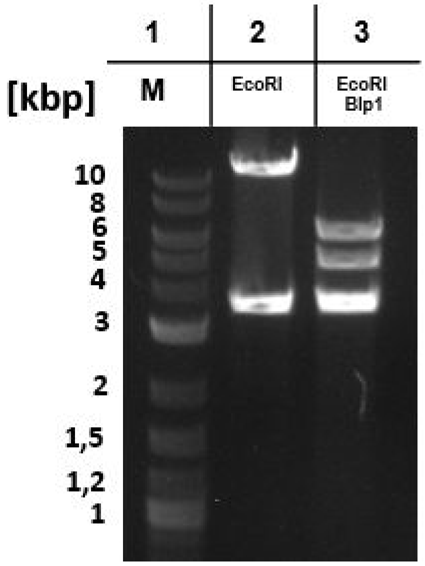

pET22

Expected Fragment size
Linearised: 11.2 kb
Double cut: 2.3 + 8.9 kb

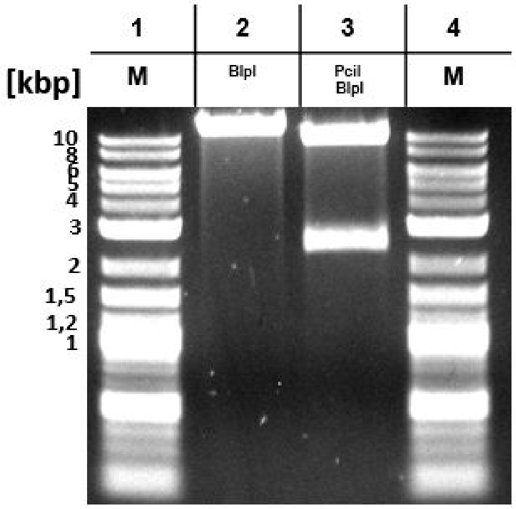

Figure S1. Agarose gels prepared to investigate the plasmid stability of the used backbones by comparing their linearized form, generated by cutting the plasmid with a compatible restriction having precisely one restriction side presents within the plasmid of interest and a fragmented version, generated by digesting the plasmid of interest with multiple restriction enzymes

.

Plasmid cards of used constructs

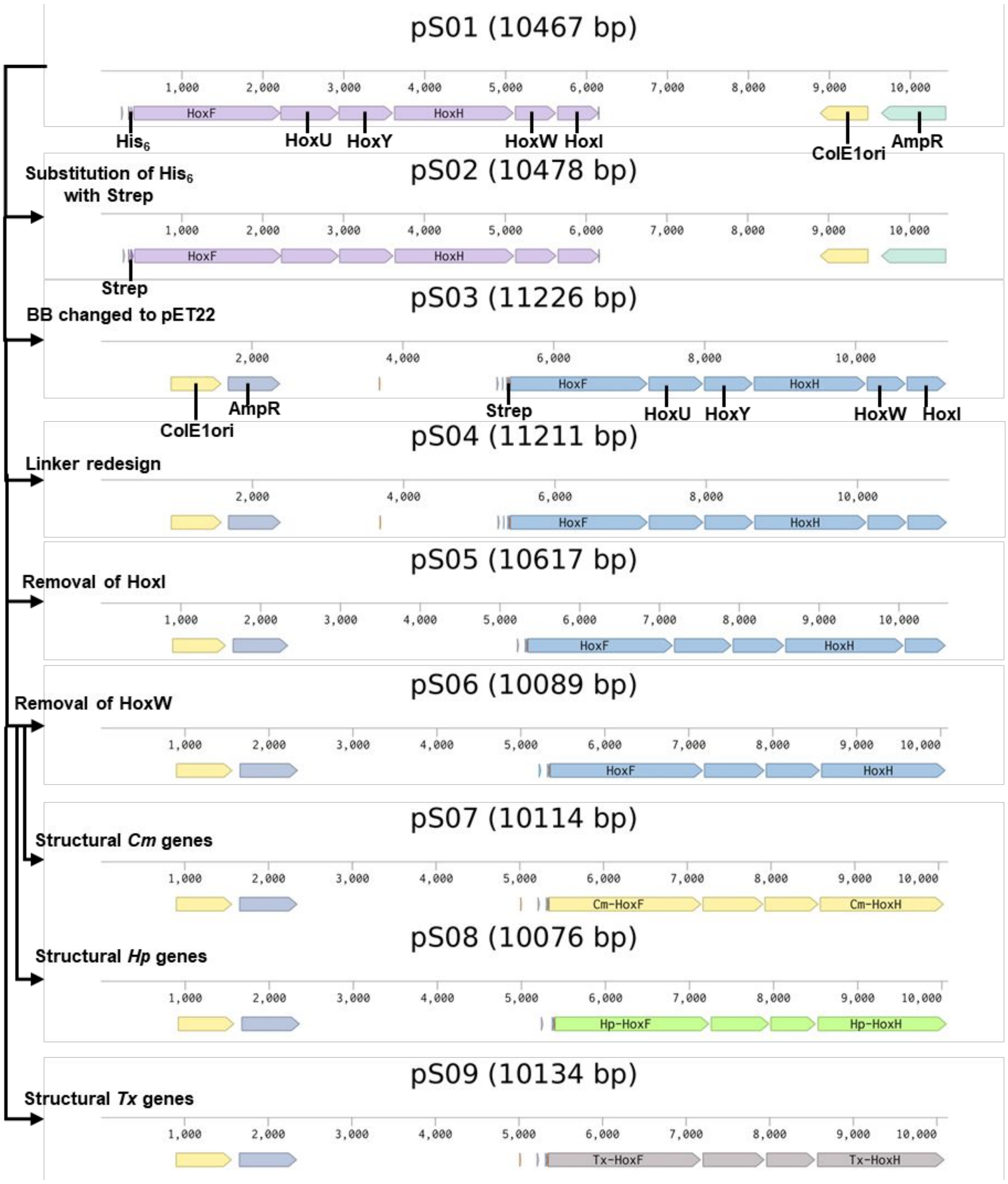

Figure S2. Plasmid cards of the constructs containing the structural genes used to produce bidirectional soluble [Ni-Fe]-hydrogenases and their individual modifications.

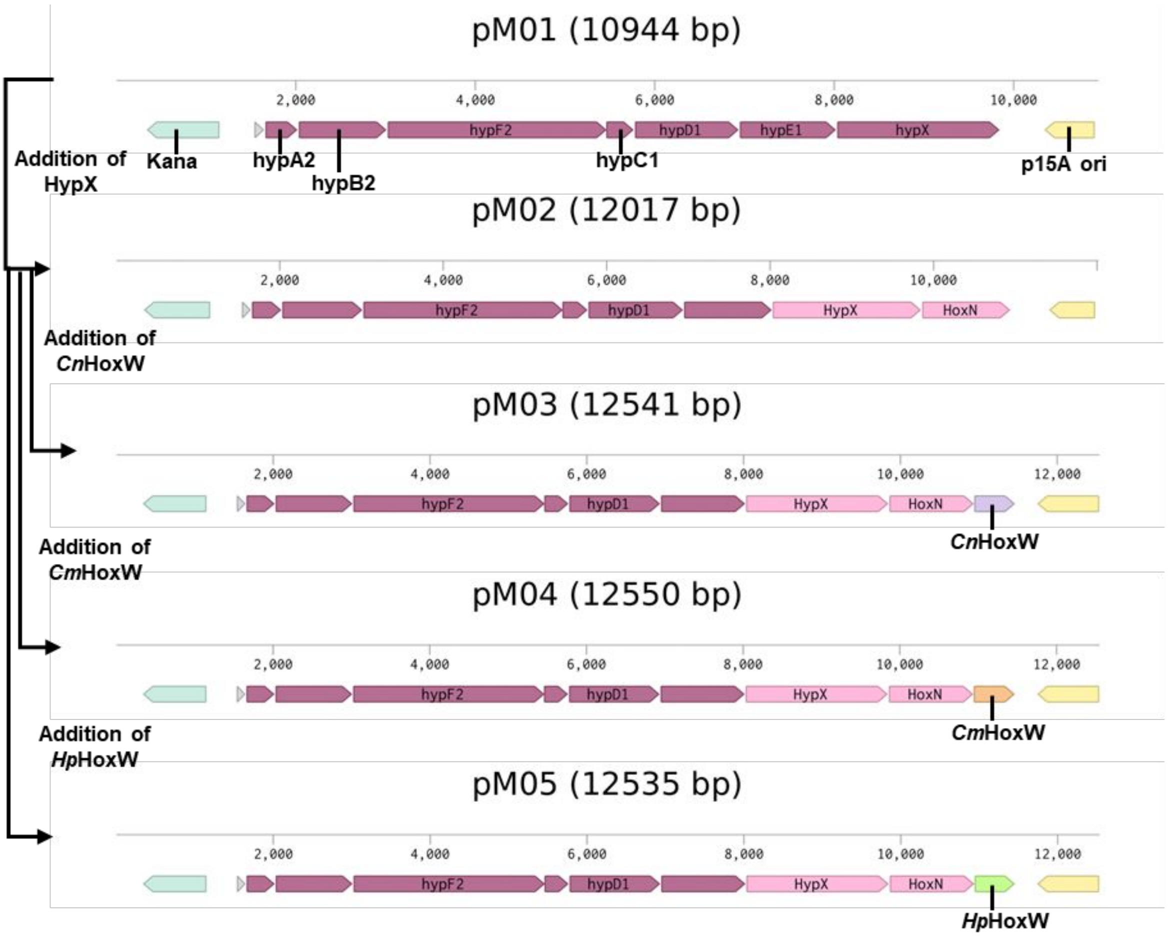

Figure S3. Plasmid cards of the constructs containing the maturational genes used to produce bidirectional soluble [Ni-Fe]-hydrogenase and their individual modifications..

Measured yields, activities and quantified coproducts

All measurements in this work were performed in biological triplicates grown and harvested according to the methods optimised the production of *Cn*SH stated in the main article. The purification of Strep-tagged enzymes was performed via Strep-TactinXT columns according to the public IBA protocol. Concentration measurements were performed via Bradford.

Table S1. Yields [mg / L of culture] of the individual purifications of soluble hydrogenases produced by the respective strains and the resulting mean and deviation

**SH produced by Plasmids**		**Purification 1 [mg/L]**	**Purification 2 [mg/L]**	**Purification 3 [mg/L]**	**Average [mg/L]**	**Deviation [mg/L]**
**PS05**	**-**	0.72	0.79	0.61	**0.71**	**0.07**
**PS05**	**PM01**	2.61	2.46	2.5	**2.52**	**0.06**
**PS05**	**PM02**	4.6	4.95	4.51	**4.69**	**0.19**
**PS06**	**PM03**	4.92	4.84	4.68	**4.81**	**0.1**
**PS02**	**PM02**	2.62	2.67	2.58	**2.62**	**0.04**
**PS03**	**PM02**	3.14	3.16	2.84	**3.05**	**0.15**
**PS04**	**PM02**	4.51	4.25	3.96	**4.24**	**0.22**
**PS07**	**PM03**	1.79	1.94	1.64	**1.79**	**0.12**
**PS08**	**PM03**	2.48	2.49	2.52	**2.5**	**0.02**
**PS09**	**PM03**	2.99	2.98	2.99	**2.99**	**0**

Table S2. NADH oxidising activity [U/mg] of the individual purifications of soluble hydrogenases produced by the respective strains and the resulting mean and deviation

**SH produced by Plasmids**		**Purification 1 [U / mg]**	**Purification 2 [U / mg]**	**Purification 3 [U / mg]**	**Average [U / mg]**	**Deviation [U / mg]**
**PS05**	**-**	0.55	0.58	0.64	**0.59**	**0.04**
**PS05**	**PM01**	0.97	0.93	0.97	**0.96**	**0.02**
**PS05**	**PM02**	1.63	1.46	1.41	**1.5**	**0.09**
**PS06**	**PM03**	1.46	1.46	1.42	**1.45**	**0.02**
**PS02**	**PM02**	0.55	0.58	0.64	**0.59**	**0.04**
**PS03**	**PM02**	0.6	0.68	0.7	**0.66**	**0.04**
**PS04**	**PM02**	1.12	1.11	1.23	**1.15**	**0.05**
**PS07**	**PM03**	0.96	0.81	0.96	**0.91**	**0.07**
**PS08**	**PM03**	1.57	1.57	1.73	**1.62**	**0.08**
**PS09**	**PM03**	0.62	0.69	0.87	**0.73**	**0.11**

Table S3. Hydrogen induced NAD^+ reduction activity [U /mg] of the individual purifications of soluble hydrogenases produced by the respective strains and the resulting mean and deviation

SH produced by Plasmids		Purification 1 [U / mg]	Purification 2 [U / mg]	Purification 3 [U / mg]	Average [U / mg]	Deviation [U / mg]
**PS05**	**-**	0.11	0.23	0.16	**0.17**	**0.05**
**PS05**	**PM01**	18.7	19.69	16.25	**18.21**	**1.45**
**PS05**	**PM02**	27.12	25.8	24.03	**25.65**	**1.27**
**PS06**	**PM03**	36.78	42.6	27.75	**35.71**	**6.11**
**PS02**	**PM02**	7.87	6.42	5.09	**6.46**	**1.14**
**PS03**	**PM02**	11.4	14.44	11.66	**12.5**	**1.38**
**PS04**	**PM02**	21.21	13.55	17.27	**17.34**	**3.13**
**PS07**	**PM03**	0.82	0.84	0.86	**0.84**	**0.02**
**PS08**	**PM03**	0.07	0.1	0.08	**0.08**	**0.01**
**PS09**	**PM03**	0	0	0	**0**	**0**
**PS07**	**PM04**	7.15	9.01	8.57	**8.24**	**0.79**
**PS08**	**PM05**	0.13	0.05	0.12	**0.1**	**0.03**
**PS09**	**PM03***	4.49	3.13	3.1	**3.57**	**0.65**
***performed und anaerobic conditions**						

Table S4. Coproduced H_2O_2 [µM per mM of converted NADH] of the individual purifications of soluble hydrogenases produced by the respective strains and the resulting mean and deviation

SH produced by Strain		Purification 1 [µM/ mM]	Purification 2 [µM/ mM]	Purification 3 [µM/ mM]	Average [µM/ mM]	Deviation [µM/ mM]
**PS05**	**-**	11.52	9.89	11.52	10.98	0.77
**PS05**	**PM01**	3.54	3.45	3.97	3.65	0.23
**PS05**	**PM02**	3.24	2.68	2.77	2.9	0.25
**PS06**	**PM03**	1.82	1.74	2.42	1.99	0.3

List of potential soluble [NiFe] hydrogenases

The following potential soluble hydrogenases were assembled based on their similarity to the respective subunits of *Cn*SH, while only considering genes from hosts, which were containing an equivalent to each of the 4 subunits. Furthermore, only candidates sharing an average similarity towards CnSH of 40% or higher were considered. The overall similarity towards the original enzyme were calculated as a mean of the individual similarities. This allowed a stronger inclusion of the smaller subunits HoxU and HoxY in contrast to an approach calculating the similarity per amino acid.

Table S5. List of potential bidirectional [NiFe]-Hydrogenases listed by their relative similarity to CnSH, accessions and structural similarities of the individual potential subunits are listed within the individual subunits candidates based on their data registered in Uniprot.

Abbrev.	native Organism	Accession HoxF	Identity to *Cn*SH HoxF[%]	Accession HoxU	Identity to *Cn*SH HoxU [%]	Accession HoxY	Identity to *Cn*SH HoxY [%]	Accession HoxH	Identity to *Cn*SH HoxY[%]	Ø Identity [%]
CnSH	Cupriavidus necator (strain ATCC 17699 / DSM 428 / KCTC 22496 / NCIMB 10442 / H16 / Stanier 337)	P22317	100	P22318	100	P22319	100	P22320	100	100
HsSH	Halomonas sp. A11-A	A0A317N828	88,4	A0A317N470	93,6	A0A317N372	92,8	A0A317N370	92,2	91,8
CmSH	Cupriavidus metallidurans (strain ATCC 43123 / DSM 2839 / NBRC 102507 / CH34)	Q1LN71	88	Q1LN70	93,6	Q1LN69	92,8	Q1LN68	92,2	91,7
SrSH	Serpentinimonas raichei	A0A060NI80	72,2	A0A060NN81	77,6	A0A060NPZ8	82,9	A0A060NIY9	82,6	78,8
MvSH	Mycolicibacterium vanbaalenii	A0A5S9R524	69,4	A0A5S9R4Y8	77,8	A0A5S9R3X3	79,3	A0A5S9R5C0	79,5	76,5
MsSH	Mycolicibacterium sp. P9-64	A0A5A7WMN9	69,2	A0A5A7WJC2	76,9	A0A5A7WIV8	79,6	A0A5A7WQB9	78,9	76,2
MrSH	Mycolicibacterium rhodesiae (strain NBB3)	G8RXY1	71,4	G8RXY0	77,3	G8RXX9	77,3	G8RXX8	78,3	76,1
AsSH	Antrihabitans stalactiti	A0A848KNW9	72,1	A0A848KVU7	72,3	A0A848KNQ3	77,6	A0A848KMX2	79,3	75,3

ObSH	Oxalobacteraceae bacterium IMCC9480	F1W2T5	71,4	F1W2T6	67,8	F1W2T7	75,9	F1W2T8	78,1	73,3
McSH	Mycobacterium cookii	A0A7I7L0H1	64,2	A0A7I7L196	77,8	A0A7I7L0Q2	70,8	A0A7I7KZK0	77,5	72,6
GsSH	Glaciimonas sp. PCH181	A0A2T6F7U3	66,1	A0A2T6F7V2	71,4	A0A2T6F7Y8	75,4	A0A2T6F819	77	72,5
MhSH	Mycobacterium helveticum	A0A557XHU4	63,7	A0A557XHT6	76,9	A0A557XHT9	70	A0A557XHT0	76,4	71,8
MdSH	Mycolicibacterium doricum	A0A1X1TBZ9	61,7	A0A1X1TBL7	77,1	A0A1X1TBK9	69,4	A0A1X1TBM9	75,6	71
GhSH	Grimontia hollisae CIP 101886	D0IAF4	59,5	D0IAF5	70	D0IAF6	76,9	D0IAF7	74,8	70,3
MpSH	Mycolicibacterium poriferae	A0A6N4VAI6	60,9	A0A6N4V920	72,6	A0A6N4V631	67	A0A6N4VAJ6	75,2	68,9
RsSH	Roseivivax sediminis	A0A1I1XWU1	62,9	A0A1I1XX75	62,4	A0A1I1XWP3	69,6	A0A1I1XWU0	75,6	67,6
NsSH	Natronogracilivirga saccharolytica	A0A8J7RL93	48,2	A0A8J7UTV3	48	A0A8J7USP9	47,8	A0A8J7RIA3	54,4	49,6
CESH	Candidatus Electrothrix aarhusensis	A0A3S3SJ71	43,3	A0A3S3QW25	48,2	A0A3S3RNE7	47,8	A0A3S3U523	54,5	48,5
FsSH	Fulvivirga sedimenti	A0A9X1HQV5	46,1	A0A9X1HNG9	46,3	A0A9X1HNG9	51,1	A0A9X1HST9	49,7	48,3
Gh2SH	Ginsengibacter hankyongi	A0A5J5IM43	46,3	A0A5J5IKL8	48,3	A0A5J5IKL8	47,2	A0A5J5IN40	51,1	48,2
BbSH	Bacteroidales bacterium	A0A942NUA2	49,1	A0A942NUS8	43,2	A0A942P0W9	45,8	A0A942S0P3	53,2	47,8
AySH	Abyssalbus ytuae	A0A9E6ZML9	43,2	A0A9E6ZLZ5	44,7	A0A9E6ZLZ5	53,2	A0A9E6ZQU4	49,7	47,7
LoSH	Lutibacter oceani	A0A3D9RXK2	45,5	A0A3D9RYW7	45,7	A0A3D9RYW7	49,4	A0A3D9RRA1	49,6	47,6
PsSH	Pontiella sulfatireligans	A0A6C2UKX6	48,1	A0A6C2UNN7	39,5	A0A6C2UKY7	51,7	A0A6C2ULA9	50,8	47,5
NvSH	Nguyenibacter vanlangensis	A0A7Y7ITF3	55,7	A0A7Y7M4I0	39,5	A0A7Y7M604	47,2	A0A7Y7M5M4	47	47,4
RbSH	Rhodothermaceae bacterium	A0A9X1HH12	45,2	A0A9X1HIA7	47,1	A0A9X1HIA7	46,3	A0A9X1HFP4	50,5	47,3
GjSH	Gramella jeungdoensis	A0A4Y8AW93	45,5	A0A4Y8AW94	42,5	A0A4Y8AW94	51,5	A0A4Y8AUV7	49,3	47,2
PdSH	Pontiella desulfatans	A0A6C2U3T6	47,5	A0A6C2U4V7	38,2	A0A6C2U3S6	52,2	A0A6C2U3F8	50,8	47,2
ZmSH	Zhengella mangrovi	A0A2G1QQD5	48,3	A0A2G1QQK7	42	A0A2G1QQH3	48	A0A2G1QQD2	50,6	47,2

AlSH	Aestuariivirga litoralis	A0A2W2AZ01	48,3	A0A2W2CFM6	41	A0A2W2CFV6	47,8	A0A2W2BFA6	50,8	47
LsSH	Lutibacter sp. B1	A0A7X8N9T8	47,8	A0A7X8N8Z2	44,5	A0A7X8N8Z2	46,1	A0A7X8NCA3	49,5	47
DsSH	Dechloromonas sp. TW-R-39-2	A0A974SYP4	43,2	A0A974SXF2	41,6	A0A974SXC7	51,9	A0A974SXB2	50,6	46,8
HcSH	Hahella chejuensis (strain KCTC 2396)	Q2SQP5	49,6	Q2SQP6	42,7	Q2SQP7	45,4	Q2SQP8	49,4	46,8
LaSH	Lutibacter agarilyticus	A0A238YY59	44,6	A0A238YXB9	43,4	A0A238YXB9	49,4	A0A238YWZ5	49,9	46,8
Lo2SH	Lutibacter oricola	A0A1H3EW96	44	A0A1H3ETS5	44	A0A1H3ETS5	49,5	A0A1H3ETN0	49,7	46,8
Ls2SH	Luteitalea sp. TBR-22	A0A915XQ57	48,8	A0A915UEZ6	42,8	A0A915UF08	42,6	A0A915UGH5	52,6	46,7
MoSH	Methylogaea oryzae	A0A8D4VPA7	46,1	A0A8D4VLS3	41	A0A8D4VN78	51,1	A0A8D5AJL7	48,5	46,7
RaSH	Roseibium aggregatum	A0A0M6Y364	46,6	A0A0M6Y1M0	43,1	A0A0M6Y4Q1	46,8	A0A0M6Y5A9	50	46,6
SlSH	Sideroxydans lithotrophicus (strain ES-1)	D5CSA5	44,3	D5CSA6	40,9	D5CSA7	51,4	D5CSA8	49,3	46,5
TsSH	Thauera sp. K11	A0A290ZT60	43,4	A0A290ZT47	39,8	A0A290ZT49	52,5	A0A290ZTC4	50,1	46,5
DdSH	Dechloromonas denitrificans	A0A133XJX5	41,6	A0A133XJ02	43,7	A0A133XIX8	51,9	A0A133XIY0	48,5	46,4
GcSH	Gallionella capsiferriformans (strain ES-2)	D9SGF2	42,5	D9SGF3	41	D9SGF4	50,6	D9SGF5	51,3	46,4
Rb2SH	Rhodobacterales bacterium	A0A5D0VDF7	46,4	A0A5D0VDE8	42,7	A0A5D0VCD8	45,9	A0A5D0VEQ4	50,6	46,4
As2SH	Azospira sp. I13	A0A2R5EE16	42,8	A0A2R5ELM5	40,7	A0A2R5EKK9	51,1	A0A2R5ECK1	50,7	46,3
Ds2SH	Dechloromonas sp.	A0A6N1XCL0	41,7	A0A6N1XA49	42,3	A0A6N1XA49	51,9	A0A6N1XDE7	49,1	46,3
MfSH	Mariprofundus ferrinatatus	A0A2K8LE31	42,5	A0A2K8L5H8	40,9	A0A2K8L5E5	51,1	A0A2K8L8H7	50,1	46,2
PcSH	Parasulfuritortus cantonensis	A0A4R1B920	41,5	A0A4R1B5X7	41,6	A0A4R1B4T9	51,4	A0A4R1B4E0	50,1	46,2
PbSH	Planctomycetes bacterium CA13	A0A5C5ZA48	46,5	A0A5C5ZCB0	41,8	A0A5C5ZA53	47,2	A0A5C5ZBF6	49,2	46,2
Ra2SH	Roseibium album	A0A0M7AXY3	46,1	A0A0M6ZK38	43,1	A0A0M7AXN4	44,9	A0A0M6ZLU2	50,6	46,2
Ts2SH	Telmatocola sphagniphila	A0A8E6B9A7	46,4	A0A8E6BAP9	41,6	A0A8E6EUB6	50	A0A8E6BAE8	46,4	46,1
TaSH	Thauera aminoaromatica	C4ZJZ5	43,8	C4ZJZ6	39,9	C4ZJZ7	51,1	C4ZJZ8	49,7	46,1

TmSH	Thiohalocapsa marina	A0A5M8FQ43	44,9	A0A5M8FRW1	39,8	A0A5M8FMX1	50,6	A0A5M8FLM4	49,1	46,1
LcSH	Lichenicola cladoniae	A0A6M8HYZ8	50,6	A0A6M8HZB7	35,7	A0A6M8HZ81	47	A0A6M8HZI0	50,5	46
Ra3SH	Roseibium alexandrii	A0A0M6ZVT8	46,2	A0A0M6ZV40	40,8	A0A0M6ZTQ8	46,8	A0A0M6ZVC1	50,2	46
AtSH	Azoarcus taiwanensis	A0A972JA32	44,4	A0A972JAQ1	43,7	A0A972J849	47,6	A0A972F7E2	48	45,9
AhSH	Azonexus hydrophilus	A0A1R1I575	42,5	A0A1R1I5J0	42,3	A0A1R1I5J2	49,4	A0A1R1I5B2	49,5	45,9
CTSH	Candidatus Thiodictyon syntrophicum	A0A2K8UD15	44,1	A0A2K8UD16	40,3	A0A2K8UD09	50,3	A0A2K8UEF7	48,8	45,9
Ds3SH	Dechloromonas sp. HYN0024	A0A3B7AH76	42	A0A3B7A8Y4	42,1	A0A3B7AE95	50,3	A0A3B7AB04	49,3	45,9
LmSH	Lutibacter maritimus	A0A1I6SL82	43,7	A0A1I6SL18	41,8	A0A1I6SL18	48,9	A0A1I6SL27	49,1	45,9
Mc2SH	Methylococcus capsulatus (strain ATCC 33009 / NCIMB 11132 / Bath)	Q603S6	44,4	Q603S5	43	Q603S4	47,2	Q60CJ2	49,1	45,9
Rs2SH	Roseibium sp. RKSG952	A0A6N8A4I6	46,7	A0A6N8A674	41,7	A0A6N8A480	44,9	A0A6N8A523	50,4	45,9
FrSH	Flavivirga rizhaonensis	A0A4S1DW70	44,2	A0A4V3P4N6	45,7	A0A4V3P4N6	44,1	A0A4S1DW95	49,2	45,8
Ms2SH	Mariprofundus sp. EBB-1	A0A498DLQ6	43,9	A0A498DLJ4	39,5	A0A498DKK7	52,2	A0A498E138	47,5	45,8
At2SH	Aromatoleum tolulyticum	A0A1N6R7A9	42,2	A0A1N6R7A1	42,2	A0A1N6R761	51,1	A0A1N6R6Y5	47,2	45,7
ArSH	Azospira restricta	A0A974PXW8	43,2	A0A974PYA1	40,4	A0A974PXT1	48,6	A0A974PXY8	50,5	45,7
gpSH	gamma proteobacterium SS-5	A0A5Q0EJ42	47,5	A0A5Q0EHS6	40,3	A0A5Q0EII7	45,4	A0A5Q0EK10	49,7	45,7
MeSH	Mariprofundus erugo	A0A5R9GPS6	41,4	A0A5R9GQT2	41,4	A0A5R9GTQ8	51,1	A0A5R9GRU1	48,7	45,7
NaSH	Novipirellula aureliae	A0A5C6EA80	45,6	A0A5C6E7U4	40,1	A0A5C6ECI4	48,3	A0A5C6E7S2	48,6	45,7
Tm2SH	Thiocapsa marina 5811	F9U760	44,6	F9U761	40,7	F9U762	49,2	F9U763	48,4	45,7
CM2SH	Candidatus Methylospira mobilis	A0A5Q0BQI4	41,8	A0A5Q0BU43	43,2	A0A5Q0BL73	48,9	A0A5Q0BLV3	48,6	45,6
DoSH	Denitratisoma oestradiolicum	A0A6S6XWV3	42,9	A0A6S6XU14	41,6	A0A6S6Y2K0	48,9	A0A6S6XYJ2	49,1	45,6
Ds4SH	Denitratisoma sp. DHT3	A0A518U7C7	42,4	A0A518U7F0	40,7	A0A518U7D6	51,7	A0A518U7E4	47,6	45,6

Mf2SH	Mariprofundus ferrooxydans PV-1	Q0EZV3	42,5	Q0EZV2	42,5	Q0EZV1	48,8	Q0EZV0	48,7	45,6
TiSH	Thioploca ingrica	A0A090BUW6	45	A0A090AL86	39,9	A0A090AFK1	51,9	A0A090ADC0	45,7	45,6
AiSH	Azospira inquinata	A0A975SMY8	40,9	A0A975SN23	41,2	A0A975SNJ2	51,1	A0A975SN11	48,9	45,5
HdSH	Hartmannibacter diazotrophicus	A0A2C9D0X5	47,3	A0A2C9D2E4	40	A0A2C9D140	47,2	A0A2C9D1B0	47,6	45,5
La2SH	Lentiprolixibacter aurantiacus	A0AAE3SP89	43,2	A0AAE3SP61	44,3	A0AAE3SP61	43,6	A0AAE3MMW8	50,9	45,5
Ls3SH	Lutibacter sp. HS1-25	A0A4Q1ILC8	40,5	A0A4Q1INR6	42,9	A0A4Q1INR6	49,2	A0A4Q1ILI8	49,2	45,5
Na2SH	Novipirellula artificiosorum	A0A5C6DEB9	46,1	A0A5C6DDE0	41	A0A5C6DGQ6	47,2	A0A5C6DEM2	47,8	45,5
TuSH	Thiothrix unzii	A0A975F8A8	41,7	A0A975F8B1	38,9	A0A975F8K6	52,2	A0A975F918	49,2	45,5
VfSH	Vitreoscilla filiformis	A0A221KE33	45,2	A0A221KEK9	39,5	A0A221KE77	46,5	A0A221KE26	50,7	45,5
CM3SH	Candidatus Methylobacter titanis	A0AA43TKJ8	42,6	A0AA43TKL0	42,9	A0AA43Q248	50	A0AA43Q1Y7	46,1	45,4
Ms3SH	Methylococcus sp. EFPC2	A0A974VWI5	42	A0A974VY98	42,6	A0A974VX12	50	A0A974VQG3	47	45,4
Ns2SH	Nitrosospira sp. Nsp14	A0A1I3B600	41,8	A0A1I3B609	44,1	A0A1I3B4L9	47,8	A0A1I3B5C1	47,7	45,4
Rs3SH	Rubrivivax sp. A210	A0A916ETG1	42,3	A0A916ACW8	43	A0A916AG24	47,8	A0A916AFX7	48,3	45,4
TgSH	Thalassovita gelatinovora	A0A0P1G2N7	46,3	A0A0N7LVX9	40	A0A0P1FHV8	46,8	A0A0P1FHU8	48,3	45,4
ZdSH	Zoogloea dura	A0A848GA31	41,8	A0A848GAB0	41,6	A0A848G697	48,6	A0A848G683	49,7	45,4
Ls4SH	Labrenzia sp. THAF82	A0A5P9CVM1	45,6	A0A5P9CVE4	41,3	A0A5P9CWN7	45,4	A0A5P9CVH4	49	45,3
Mo2SH	Methylocucumis oryzae	A0A0F3IHC5	42,7	A0A0F3IHG9	41,5	A0A0F3IHN6	50,6	A0A0F3IK14	46,5	45,3
SsSH	Sulfuriferula sp. AH1	A0A1Y0GBD0	43,2	A0A1Y0G9M6	41,3	A0A1Y0G956	48,9	A0A1Y0G9B6	47,8	45,3
TpSH	Thauera propionica	A0A235EWZ2	42,8	A0A235EW75	39,2	A0A235EWD2	48,9	A0A235EWB1	50,1	45,3
ZoSH	Zoogloea oleivorans	A0A6C2CKY5	41,6	A0A6C2CMK1	41,4	A0A6C2CK41	49	A0A6C2CKU7	49,3	45,3
As3SH	Azoarcus sp. (strain BH72)	A1K5C4	43,2	A1K5C5	40,1	A1K5C6	48,9	A1K5C7	48,4	45,2
Ns3SH	Novosphingobium sp. FSW06-99	A0A117UW65	49,1	A0A117UWA0	37,3	A0A117UW57	45,6	A0A117UW95	48,8	45,2
Pc2SH	Parazoarcus communis SWub3 = DSM 12120	A0A323V6Y7	41,5	A0A323UU04	42,9	A0A323UXC6	47,2	A0A323UTR9	49,1	45,2

PlSH	Pseudothauera lacus	A0A2T4IFD8	41,2	A0A2T4IFC1	42,2	A0A2T4IFB8	48,7	A0A2T4IFE0	48,7	45,2
Ts3SH	Thauera sp. 27	N6XKD0	42,7	N6YIG9	40,8	N6YIX6	48,1	N6YQ70	49,3	45,2
CM4SH	Candidatus Methylobacter favarea	A0A8S0YAB2	42,7	A0A8S0X8T2	42,7	A0A8S0WB77	49,4	A0A8S0XH20	45,5	45,1
Mh2SH	Methylocystis heyeri	A0A6B8KK41	46,8	A0A6B8KL31	35	A0A6B8KKA0	51,2	A0A6B8KIZ5	47,3	45,1
SvSH	Solemya velesiana gill symbiont	A0A1T2KWB5	42,5	A0A1T2KWC9	40,7	A0A1T2KWE3	47,8	A0A1T2KWM2	49,2	45,1
ShSH	Sulfuritalea hydrogenivorans sk43H	W0SGG0	41,7	W0SFQ3	40,5	W0SFZ3	47,8	W0SIU8	50,5	45,1
AfSH	Albidiferax ferrireducens (strain ATCC BAA-621 / DSM 15236 / T118)	Q21RP6	43	Q21RP7	43,5	Q21RP8	45,1	Q21RP9	48,2	45
CT2SH	Candidatus Thiothrix singaporensis	A0A7L6AWT9	41,3	A0A7L6AX04	40	A0A7L6AX04	51,7	A0A7L6AXC7	47	45
Ms4SH	Methylocella silvestris (strain DSM 15510 / CIP 108128 / LMG 27833 / NCIMB 13906 / BL2)	B8ERX4	49,9	B8ERX3	36,8	B8ERX2	44,4	B8ERX1	48,9	45
MiSH	Methylomagnum ishizawai	A0A1Y6CSE5	41,7	A0A1Y6CTL2	42,1	A0A1Y6CSZ2	48,9	A0A1Y6CYN9	47,2	45
Mp2SH	Methylomonas paludis	A0A975RA95	44	A0A975R944	41	A0A975RAB3	48,9	A0A975MNH6	46	45
Rb3SH	Rhodocyclaceae bacterium	A0A2K9LBI2	43	A0A2K9L919	41,9	A0A2K9L9E1	46,4	A0A2K9L8V4	48,8	45
As4SH	Azoarcus sp. KH32C	H0PVJ4	41,6	H0PVJ3	41,3	H0PVJ2	49,4	H0PVJ1	47,3	44,9
NcSH	Nitrosomonas communis	A0A0F7KDG6	41,7	A0A0F7KKG9	41,2	A0A0F7KEE5	49,4	A0A0F7KJ72	47,1	44,9
NnSH	Nitrosomonas nitrosa	A0A1I4KVK6	41,3	A0A1I4KUZ0	40	A0A1I4KVM5	50,6	A0A1I4T177	47,5	44,9
Sv2SH	Solemya velum gill symbiont	A0A0B0H874	40,7	A0A0B0HCW3	39,7	A0A0B0H9I2	51,4	A0A0B0H527	47,8	44,9
SdSH	Sulfuricella denitrificans (strain DSM 22764 / NBRC 105220 / skB26)	S6AL80	40,8	S6ACB0	38,6	S6B471	50,5	S6AH41	49,5	44,9

Ss2SH	Sulfurisoma sediminicola	A0A497X8Z9	40,2	A0A497X886	41	A0A497X7U7	50	A0A497X7H2	48,2	44,9
CsSH	Cycloclasticus sp. (strain P1)	K0C0P6	41	K0C2W5	42,2	K0C542	47,8	K0C599	48	44,8
Hs2SH	Hahella sp. CCB-MM4	A0A261GN60	46,8	A0A261GN61	39,6	A0A261GQD5	42,7	A0A261GPH8	50	44,8
Ms5SH	Magnetospirillum sp. SS-4	A0A6J4FGE0	41,6	A0A6J4FGN2	36,4	A0A6J4FHN2	51,7	A0A6J4FGG6	49,3	44,8
PmSH	Paramagnetospirillum marisnigri	A0A178MPA5	41,8	A0A178MP00	36,3	A0A178MMG5	50,5	A0A178MM77	50,5	44,8
CPSH	Candidatus Propionivibrio aalborgensis	A0A1A8XF33	39,2	A0A1A8XDR0	38,3	A0A1A8XHB6	53	A0A1A8XDR3	48,4	44,7
Mh3SH	Marivita hallyeonensis	A0A1M5WG78	47,3	A0A1M5WHC7	38,7	A0A1M5WGP5	44,3	A0A1M5WGL0	48,4	44,7
MtSH	Methylobacter tundripaludum (strain ATCC BAA-1195 / DSM 17260 / SV96)	G3IU92	42,2	G3IU91	42,3	G3IU90	47,8	G3IU89	46,5	44,7
MlSH	Methylomonas lenta	A0A177MY67	42,5	A0A177MYH7	41,5	A0A177MZ15	48,9	A0A177MZD3	46	44,7
Pc3SH	Parazoarcus communis	A0A2U8GV00	41,7	A0A2U8GQS3	41,4	A0A2U8GMV7	48,3	A0A2U8GNU6	47,3	44,7
Rs4SH	Rhizobacter sp. AJA081-3	A0A975E1X1	43,5	A0A975E2U8	41,6	A0A975E6E6	43,9	A0A975E2Q5	49,7	44,7
ZrSH	Zoogloea ramigera	A0A4Y4CVM3	42,6	A0A4Y4D2G6	38,8	A0A4Y4CZ17	49,4	A0A4Y4CVN8	47,8	44,7
Ns4SH	Nitrosomonas sp. Nm166	A0A1I1XBV7	40,4	A0A1I1XBS2	41,5	A0A1I1XB47	48,9	A0A1I1XGM9	47,7	44,6
NmSH	Nitrosospira multiformis (strain ATCC 25196 / NCIMB 11849 / C 71)	Q2Y8F3	43,1	Q2Y8F2	39,8	Q2Y8F1	46,5	Q2Y8F0	49,1	44,6
PrSH	Pseudothauera rhizosphaerae	A0A4S4AS15	42,2	A0A4S4ARW1	41,7	A0A4S4AT42	45,8	A0A4S4AS19	48,6	44,6
StSH	Sedimenticola thiotaurini	A0A0F7K0X7	41,2	A0A0F7JVB5	39,2	A0A0F7JXT1	48,3	A0A0F7K0I7	49,5	44,6
Ss3SH	Sulfuricella sp.	A0A942PQE9	42,4	A0A942PSG7	38,4	A0A942PYP9	48,3	A0A942PFQ5	49,1	44,6
Ar2SH	Accumulibacter regalis	A0A011R2Y7	39	A0A011Q8R1	39,2	A0A011PD67	52,2	A0A011Q8J4	47,6	44,5
Ms6SH	Methylomonas sp.	A0A2P5N778	41,9	A0A2P5N6Z8	39,6	A0A2P5N6Y4	50,6	A0A2P5N6X1	46	44,5

Ms7SH	Methyloprofundus sp.	A0A7R7AGF8	41,1	A0A7R7AIF3	42,3	A0A7R7AFP3	47,8	A0A7R6W923	46,9	44,5
NhSH	Nitrosomonas halophila	A0A1H3ESH6	42,5	A0A1H3ESG3	40,6	A0A1H3ESU8	48,9	A0A1H3ESH8	45,8	44,5
CASH	Candidatus Accumulibacter sp. BA-94	A0A011QQ06	39,7	A0A011RJ19	41,2	A0A011P885	49,4	A0A011QPE6	47,1	44,4
CN2SH	Candidatus Nitrotoga fabula	A0A916F9R7	41,7	A0A916FB52	38,2	A0A916FCM7	47,8	A0A916BCH5	49,9	44,4
CS2SH	Candidatus Sedimenticola endophacoides	A0A6N4DXN6	43,7	A0A657PTL1	39	A0A657PMZ8	47,8	A0A6N4E1Y5	47	44,4
MmSH	Methylomonas methanica (strain DSM 25384 / MC09)	F9ZZP0	41,9	F9ZZN9	39,4	F9ZZN8	48,9	F9ZZN7	47,2	44,4
Ms8SH	Methylomonas sp. LL1	A0A7T0JL82	41,6	A0A7T0JM44	40,2	A0A7T0JLW3	48,9	A0A7T0JLA1	47	44,4
NlSH	Nitrosospira lacus	A0A1W6SST7	42,3	A0A1W6STQ5	38,6	A0A1W6SSW2	48,9	A0A1W6SSU1	47,7	44,4
Sl2SH	Sulfurimicrobium lacus	A0A6F8VFS5	41,1	A0A6F8VF68	39,2	A0A6F8VEN0	47,3	A0A6F8VGF2	49,9	44,4
TxSH	Tepidicella xavieri	A0A4R6U8I9	41,9	A0A4R6UCW4	39,2	A0A4R6U7N4	47,4	A0A4R6U5U3	49,1	44,4
DiSH	Denitromonas iodatirespirans	A0A944H9N7	41,8	A0A944H9V5	38,8	A0A944HAM9	47,3	A0A944HFD2	49,3	44,3
GtSH	Georgfuchsia toluolica	A0A916N986	41,8	A0A916NHN8	36,6	A0A916N8R8	48,6	A0A916J538	50,1	44,3
Ms9SH	Methylomonas sp. (strain DH-1)	A0A172UC21	42,4	A0A172UBX2	40,9	A0A172UBB3	46,7	A0A172UBD3	47	44,3
Na3SH	Nitrosomonas aestuarii	A0A1I3ZSL2	42,9	A0A1I3ZS69	40,1	A0A1I3ZSN9	46,1	A0A1I3ZSQ8	48,1	44,3
CbSH	Comamonadaceae bacterium G21597-S1	A0A9X3GP45	42,7	A0A9X3GUY0	40,8	A0A9X3GNS2	46,4	A0A9X3GQ69	46,7	44,2
Ms10SH	Magnetospirillum sp. ME-1	A0A1W6CHE8	40,1	A0A1W6CHE4	35,1	A0A1W6CHD4	50,6	A0A1W6CHE2	50,9	44,2
MaSH	Methylotuvimicrobium alcaliphilum (strain DSM 19304 / NCIMB 14124 / VKM B-2133 / 20Z)	G4SW81	42,7	G4SW80	38,9	G4SW79	48,9	G4SW78	46,1	44,2
Mp3SH	Methylovulum psychrotolerans	A0A1Z4BUB3	42,3	A0A1Z4BUE9	39,3	A0A1Z4BU82	47,8	A0A1Z4BU24	47,1	44,1

OdSH	Oharaeibacter diazotrophicus	A0A4R6R6V0	42,4	A0A4R6R6G2	40,4	A0A4R6R6Q9	48,1	A0A4R6R6F2	45,6	44,1
Ra4SH	Rhodobacter aestuarii	A0A1N7L0B7	41,9	A0A1N7KZS9	42,6	A0A1N7KZR0	45,6	A0A1N7KZN8	46,1	44,1
Ts4SH	Thiohalocapsa sp. PB-PSB1	V4IX68	43,4	V4JGW4	39,4	V4JRQ7	47,1	V4JK56	46,4	44,1
Md2SH	Methylomonas denitrificans	A0A140E5B2	41,9	A0A140E5B3	38,9	A0A140E5B4	49,4	A0A140E5B5	45,6	44
Ms11SH	Methylomonas sp. LWB	A0A1S1Y5W2	41,4	A0A1S1Y6M9	38,3	A0A1S1Y665	49,4	A0A1S1Y621	46,8	44
Nm2SH	Nitrogeniibacter mangrovi	A0A6C1B645	41	A0A6C1B4C9	40,8	A0A6C1B254	47,5	A0A6C1B2C3	46,8	44
NuSH	Niveibacterium umoris	A0A840BHB4	40,5	A0A840BDM6	42,1	A0A840BF23	46,4	A0A840BJ72	46,9	44
Cp2SH	Chitinilyticum piscinae	A0A8J7FM65	42,3	A0A8J7K7Y9	39,7	A0A8J7FJM7	46,7	A0A8J7K162	46,8	43,9
Ma2SH	Methylomicrobium album BG8	H8GHZ0	41,2	H8GHY9	41,5	H8GHY8	46,7	H8GHY6	46	43,9
Ms12SH	Methyloprofundus sedimenti	A0A1V8MAA8	41,7	A0A1V8MA80	40,5	A0A1V8MA78	46,7	A0A1V8M8T6	46,7	43,9
NoSH	Nitrosomonas oligotropha	A0A1H8KSX4	39,8	A0A1H8KSZ8	38,6	A0A1H8TNT6	50	A0A1H8TMF2	47,2	43,9
PxSH	Paraburkholderia xenovorans (strain LB400)	Q13HL1	41,8	Q13HL0	40,2	Q13HK9	47,8	Q13HK8	45,8	43,9
CA2SH	Candidatus Accumulibacter cognatus	A0A080M8N1	39,3	A0A080MA64	37,8	A0A080MJB4	50,3	A0A080M7X3	47,6	43,8
IdSH	Ideonella dechloratans	A0A643FF27	42,5	A0A643FG42	36,9	A0A643FFH6	46,2	A0A643FHM1	49,7	43,8
Lc2SH	Leptothrix cholodnii (strain ATCC 51168 / LMG 8142 / SP-6)	B1Y7Y8	42,2	B1Y7Y7	39,8	B1Y7Y6	45	B1Y7Y5	48,2	43,8
CA3SH	Candidatus Accumulibacter adjunctus	A0A011NYC2	39,9	A0A011MI88	40,1	A0A011PTF4	48,3	A0A011NYE4	46,1	43,6
TdSH	Thiobacillus denitrificans	A0A106BJG8	41,8	A0A106BJ90	39,6	A0A106BJ91	43,9	A0A106BJ79	48,9	43,6
CA4SH	Candidatus Accumulibacter aalborgensis	A0A1A8XKB7	39,9	A0A1A8XLS7	38,7	A0A1A8XMJ0	48,2	A0A1A8XKC2	47,2	43,5

CS3SH	Candidatus Symbiobacter mobilis CR	U5N6R8	41,1	U5N6C1	38,6	U5N705	46	U5NA59	47,8	43,4
Ms13SH	Methyloversatilis sp. RAC08	A0A1B3LP78	41,4	A0A1B3LKX1	36,2	A0A1B3LS33	46,4	A0A1B3LLR7	48,7	43,2
Rs5SH	Rhodovulum sp. PH10	J6LIG2	39,5	J6UGV0	38,9	J6JAZ3	46,1	J6UFT5	47,7	43,1
Ta2SH	Thauera aromatica K172	A0A2R4BNY7	41,3	A0A2R4BNT0	37,5	A0A2R4BNI6	45,6	A0A2R4BNM7	47,4	43
Hs3SH	Hydrogenophaga sp. A37	A0A1V3SGG3	41,7	A0A1V3SER0	35,9	A0A1V3SF10	47,7	A0A1V3SEV3	46	42,8
MbSH	Methylocystis bryophila	A0A1W6N1X5	41,5	A0A1W6MZ65	37,7	A0A1W6MZ81	45,6	A0A1W6MZB9	46,2	42,8
HaSH	Hydrogenophaga aromaticivorans	A0A7Y8GY12	40,9	A0A7Y8GYN8	35,1	A0A7Y8KYT4	46,7	A0A7Y8KZB3	47,6	42,6
EbSH	Ectothiorhodospiraceae bacterium BW-2	A0A5C2HJW2	39,7	A0A5C2HJW2	39,5	A0A5C2HSJ8	44,4	A0A5C2HJR3	46,3	42,5
Ra5SH	Rubrivivax albus	A0A437JST4	42	A0A3S2U7J0	38,7	A0A3S2TPP4	43,3	A0A3S2VVQ4	46	42,5
IaSH	Ideonella aquatica	A0A940YIP1	42	A0A940YGJ3	38,2	A0A940YCR3	44,9	A0A940YH69	44,6	42,4
CcSH	Coleofasciculus chthonoplastes PCC 7420	B4VSZ9	39,7	B4VT00	40,9	B4VT01	44,7	B4VT02	43,3	42,2
PiSH	Psychromonas ingrahamii (strain DSM 17664 / CCUG 51855 / 37)	A1SU84	38,4	A1SU83	34,7	A1SU83	51,7	A1SU82	44,1	42,2
Hs4SH	Hydrogenophaga sp. H7	A0A1V4CDR9	41,5	A0A1V4CDY4	35,5	A0A1V4CDS2	45	A0A1V4CDZ7	45,8	42
MgSH	Merismopedia glauca CCAP 1448/3	A0A2T1C3J9	43	A0A2T1C3M4	40,8	A0A2T1C397	40	A0A2T1C395	44,3	42
Ms14SH	Motiliproteus sp. MSK22-1	A0A1R1LYX5	39,4	A0A1R1LYW6	40,9	A0A1R1LYX8	45,6	A0A1R1LYV6	41,8	41,9
HtSH	Hydrogenophilus thermoluteolus	A0A077L6X8	40	A0A077L885	36,4	A0A077L7R5	44,4	A0A077LAI5	46,3	41,8
AoSH	Aquabacterium olei	A0A2U8FVD2	43,9	A0A2U8FTG1	35,2	A0A2U8FTP2	39,8	A0A2U8FT20	48	41,7
As5SH	Aquabacterium sp. NJ1	A0A0A0DWK0	40,7	A0A0A0DTQ2	36,3	A0A0A0DW27	43,1	A0A0A0DRK6	46,7	41,7
Cs4SH	Coleofasciculus sp. LEGE 07092	A0A929AU22	40,8	A0A929ATL2	38,4	A0A929ARE3	42,6	A0A929ARU0	44,5	41,6

PtSH	Planktothrix tepida PCC 9214	A0A1J1LPW9	40,8	A0A1J1LR17	38,9	A0A1J1LPM1	40,8	A0A1J1LRJ5	45,3	41,5
SmSH	Sphaerotilus montanus	A0A7Y9R3V3	39,4	A0A7Y9R1X6	36,5	A0A7Y9R1X6	44,2	A0A7Y9R0R6	45,7	41,5
Ls5SH	Lyngbya sp. (strain PCC 8106)	A0YSR0	40,2	A0YSQ8	36,5	A0YSQ7	44,1	A0YSQ6	44,4	41,3
Af2SH	Aerosakkonema funiforme FACHB-1375	A0A926VEP2	42,2	A0A926VH50	35,5	A0A926VF25	42	A0A926VEL4	44,1	41
Hs5SH	Hydrocoleum sp. CS-953	A0A261KJL9	41,6	A0A261KJY6	37,5	A0A261KJZ3	41,8	A0A261KK26	43,2	41
PaSH	Planktothrix agardhii (strain NIVA-CYA 126/8)	A0A073CHS6	40,2	A0A073CIW8	37,4	A0A073CIB3	42,3	A0A073CV30	44,2	41
Ls6SH	Limnothrix sp. P13C2	A0A1C0VDK4	43,9	A0A1C0VDF5	36,1	A0A1C0VDL9	37	A0A1C0VDE3	46,7	40,9
TlSH	Thermovirga lienii (strain ATCC BAA-1197 / DSM 17291 / Cas60314)	G7V5R9	43,5	G7V700	33,3	G7V7T4	42,2	G7V7T3	44,5	40,9
TtSH	Thiobaca trueperi	A0A4R3MSL2	42,7	A0A4R3N468	32,2	A0A4R3N4Z7	46,9	A0A4R3N322	41,8	40,9
Pc4SH	Pleurocapsales cyanobacterium LEGE 06147	A0A929B5E4	40,7	A0A929B3P9	36	A0A929B6P9	41,2	A0A929B5P6	45,4	40,8
At3SH	Anaerolinea thermophila (strain DSM 14523 / JCM 11388 / NBRC 100420 / UNI-1)	E8N5U2	41,8	E8N4C3	32,1	E8N3I7	43,2	E8N3I6	45,5	40,7
Ps2SH	Pelagibaculum spongiae	A0A2V1GRM0	35	A0A2V1GTM5	38,7	A0A2V1GPU3	46,7	A0A2V1GXY9	41,8	40,6
AbSH	Anaerolineales bacterium	A0A916N501	38,2	A0A916IBE5	31,9	A0A916GC14	41,8	A0A916N119	50	40,5
Af3SH	Allocoleopsis franciscana PCC 7113	K9WG92	40,8	K9WH13	37,4	K9WF30	40,6	K9WGB6	42,8	40,4
IpSH	Imhoffiella purpurea	W9VG04	42,1	W9VBK8	32,2	W9VC12	45,8	W9VIZ7	41,5	40,4
KaSH	Ketobacter alkanivorans	A0A2K9LQF6	37,9	A0A2K9LQF1	36,4	A0A2K9LQV5	45	A0A2K9LQK8	42,2	40,4

DtSH	Dictyoglomus turgidum (strain DSM 6724 / Z-1310)	B8DZB6	44	B8DYN2	32,3	B8DYN3	40,1	B8DYN4	44,4	40,2
OcSH	Oscillatoriales cyanobacterium USR001	A0A1C0VRI8	41,6	A0A1C0VRJ5	36,5	A0A1C0VRJ4	40	A0A1C0VRI9	42,8	40,2
OhSH	Okeania hirsuta	A0A3N6QP13	40,3	A0A3N6NZI4	37,2	A0A3N6NNB8	39,7	A0A3N6QWC6	42,6	40
Ps3SH	Pleurocapsa sp. PCC 7327	K9T019	36,5	K9T500	36,5	K9T7I5	43,5	K9T602	43,3	40

Cell free lysate activities of screened soluble hydrogenases

The Production of cell free lysate used for this screening was started by transforming the desired construct into BL21(DE3) - *E.coli* cells and growing them on LB plates containing the desired antibiotics. Subsequently, a single colony was used to start a preculture in LB containing the same antibiotics this culture was subsequently used to produce a main culture of 50 mL TB media containing appropriate antibiotics as well as 100 µM of Ni^{2+} and Fe^{3+} . This culture was grown at 37°C until an optical density of 2 was reached, subsequently cooled to 25 °C and induced with 1 mM of IPTG. After 16h of protein expression, the culture was harvested by centrifugation at 10000 g and 4°C and frozen for at – 80°C for at least 18h. The resulting pellet was resuspended in a suitable reaction buffer, lysed by sonification and remaining cell fragments were removed by centrifugation at 45000 g and 4 °C. The resulting cell free lysate was diluted to a protein concentration of 1 mg / ml and applied in further assays. To measure the Hydrogen-driven NAD^+ reduction in Lysate, ROTILABO® glass cuvettes were filled with 1.8 mL of 1.11 times concentrated reaction buffer, which was sealed inside and subsequently saturated with Hydrogen for a least 15 minutes. Afterwards, the reaction was initiated by adding 200 µL of cell free lysate to reach final reaction conditions of 50 mM Tris HCl, 1 mM NAD^+, 1 mM TCEP and 1 µM FMN at pH 8.

Table S6. Hydrogen induced NAD^+ reduction activity [U /g] of the individual cell free lysate samples of soluble hydrogenases produced by the respective strains and the resulting mean and deviation

SH produced by plasmids		Lysate activity 1 [U/ g of total protein mass]	Lysate activity 2 [U/ g of total protein mass]	Lysate activity 3 [U/ g of total protein mass]	Average [U/ g of total protein mass]	Deviation [U/ g of total protein mass]
	**PM03**	1.5	1.5	2	1.67	0.24
**PS07**	**PM03**	7.75	13.14	12.92	11.27	2.49
**PS08**	**PM03**	12.98	14.57	14.71	14.08	0.78
**PS09**	**PM03**	15.8	26.82	20.5	21.04	4.51

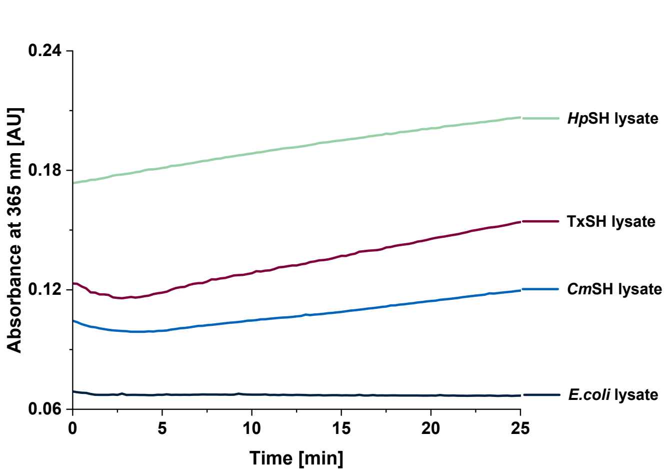

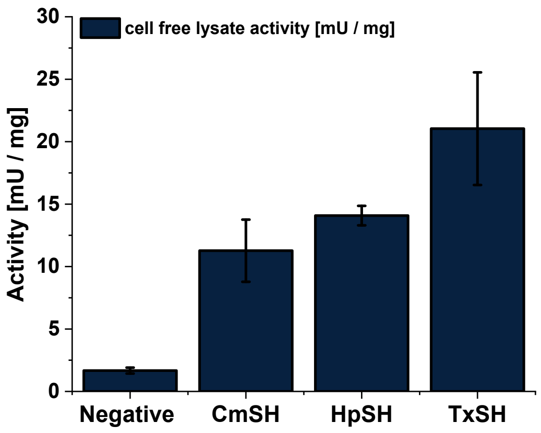

Figure S4.A) Graph presenting the hydrogen induced NAD^+ conversion by cell free lysate of the individual potential hydrogenases compared to the background reaction generated by using lysate from E.coliBL21 expressing only the maturation genes utilised for SH production and the calculated activities based on these graph(B).

Media compositions

Table S7. Composition of medias used in this study

Name of the media	Tryptone [g/L]	Yeast extract [g/L]	NaCl [g/L]	Additional components
**LB-media**	10	5	10	
**LB-Agar**	10	5	10	15 g/L of agar
**TB-media**	12	24	0	1 % glycerol (v/v)<br>17µM KH_2PO_4<br>72µM K_2HPO_4

Utilised primers

Table S8. List of utilised primers

Name	Sequence	Utilised for
**P1**	AAGCTTGTCGACCAATTGTTATTAAGAACGTTTAC	Insertion of HoxN
**P2**	GGCGTAATCATGGTCATAGCTGTTTCC	Insertion of HoxN
**P3**	ATAATAAGATCTGAGCAGGAGGAAAAAAAATGAACGCGCCGGCGG	Transfer of HoxW
**P4**	TTTTTTTCCTCCTGCTCAGATCTTATTATGCACGAACTTCGCTATCATCATAACGA	Transfer of HoxW
**P5**	ACAGGAAACAGCTATGACCATGATTACGCCTTATTAAGAGGTCTGACGTTCCGCACG	Transfer of HoxW
**P6**	GCGGGTGGCTCCACGATCCTCTCATAGTTAATTTCTCCTCTTTAATGAATTCTG	Changed His_6 to Strep
**P7**	GCCACCCGCAGTTCGAAAAAGGATCCAGGAGGAATTAATAATGG	Changed His_6 to Strep
**P8**	ATGTATATCTCCTTCTTAAAGTTAAACAAAATTATTTCTAGAGGGGA	Amplifying pET22 Backbone
**P9**	TGAGATCCGGCTGCTAACAAAGC	Amplifying pET22 Backbone
**P10**	CTTTCGGGCTTTGTTAGCAGCCGGATCTCATTATTATGCACGAACTTCGCTATCATCATAACGAC	Amplifying CnSH Insert
**P11**	TTTTGTTTAACTTTAAGAAGGAGATATACCATGCACGAAATGTCTCTGGCGG	Amplifying CnSH Insert
**P12**	CGCACCTTTTTCGAACTGCGGGTGGCTC	Linker modification
**P13**	GAAAAAGGTGCGATGGACTCTCGTATCACCACCATCCT	Linker modification

Sequence of additional enzymes

The novel enzymes introduced in this work are listed as ***Cm*SH**, ***Hp*SH** and ***Tx*SH** in in table S5 for easier accessibility, the primary sequence of these enzymes are individual provided below.

*Cm*SH

HoxF:

MSKDIRTILERYRSDRARLMDILWDVQHLYGHIPDEVLPQLAAELNLSPLDIRETASFYHLFHDKPSGKH
RIYLCNSVIAKMNGYQAVHDALERETGVRFGETDPNGMFGLFETPCIGLSDQEPAMLIDKVVFTRLRPG
KIADIIAQLKQGRSPAEIANPAGLPSDDIAYVDALVESNVRTKGPVFFRGRTDFKAVLDHCLTLRPEQVI
DEIIESKLRGRGGAGFSTGLKWQLCRRALSDTKYVICNADEGEPGTFKDRALLTRSPKEVFIGMAIAAH
AIGCRHGIVYLRAEYFYLKDYLERQLQQLRDDGLLGRAIGVRRDFDFDIRIQMGAGAYICGDESALIESC
EGKRGTPRVKPPFPVQQGYLGKPTCVNNVETFAAVSRIMEEGADWFRAMGTPDSAGTRLLSVAGDCS
KPGIYEVEWGVTLNEVLAMVGAKEARAVQISGPSGECVSVEKDGERRLAYEDLSCNGAFTIFNRNRDL
LDIVKDYMQFFVDESCGICVPCRAGNVDLHRKVEWVIAGKACQKDLDDMVSWGALVRKTSRCGLGA
TSPKPILTTLEKFPEIYQDKLVRHEGPLLPSFDLDTALGGHEKALKELEEAKK**

HoxU:

MSIQITIDGITVTTEEGRTLVDVAAENGVYIPTLCYLKDKPCLGTCRVCSVKVNGNVNAACTVRVSKGL
NVEVNSPEMVDMRKALVEFLFAEGNHNCPSCEKSGRCQLQAVGYEVDMMVSRFPYRFPVRVQDQASE
KIWLERDRCIFCQRCVEFIRDKATGRKIFSISQRGSESRIEIDVELANAMPPEQVKEAVAICPVGTILEKRV
GYDDPIGQRKYEIQSVRARALGLEGVDK**

HoxY:

MSTAAKNELASHELPATPMDPALAANREGKIKVSMIGLCGCWGCTLSFLDMDERLLPLLEKITILRSSLT
DIKRIPERCAIGFVEGGVANEENIETLEHFRENCDILISVGACAVWGGVPAMRNVVELKDCLAEAYVNS
ATAVAGAKAVIPFHPDIPRITTKVYPCHEVVKMDYFIPGCPPDGDAIFKVLDDLVNGRPFDLPSSINRYD*
*

HoxH:

MSRKLVIDPVTRIEGHGKVTVHLDDDNNVIDAKLHVVEFRGFEKFIQGHPYWEAPMFLQRICGICFVSH
HLCGAKALDDMVGVGLKSGIDVTPAAEKMRRLGHYAQMLQSHTTAYFYLIVPEMLFGMDAPPEQRN
VLGLIEADPELVKRVVMLRKWGQEVIKVVFGKKMHGINSVPGGVNKNLSIAERDRLLNGEEGLLAMD
QVIDFAQDGLRLFYDFHEKHRAQVDSFADVPALNMSLVDADGNVDYYHGKLRIVDDDKNIVRELDYH
DYLDHFSEAVEEWSYMKFPYLKDLGREKGSVRVGPLGRMNVTKTLSTPLAQEALERFHAYTKGRANN
MTLHTNWARAIEILHAAEVIRELLHDPDLQKDQLVLTPPAGAWTGEGVGVVEAPRGTLLHHYRADER
GNITFANLVVATTQNNQVMNRTVRSVAEDYLGGHGEITEGMMNAIEVGIRAYDPCLSCATHALGQMP
LVVSVYDAAGGLIDERTR**

***Hp*SH**

HoxF:

MTAFDQPSALHAADAVLARWQHDPHALVQVLRETQAQTHWLPRELLAHIAAALRLTTAHVEGVASFY
RFFHLRPVGRVHVLFSDNITDRMAGSDALMARLCARLGVAPGQVDAQGRFCVDRCSCTGLCDQGPAL
LVNHHQVVTRLDAGRVDQLAERLLAGVPPEDWPADWFAVEDHVRRADVLLQGLPADAPSLPAVLSRT
PAELLAEVERSGLRGRGGAGFPTARKWRACAEADAPDGQRCVVCNADEGEPGTFKDRVLLGRHADEL
FDGMTLAARAIGAQTGLVYLRGEYRFLLPQLEAVLQRRRANGLLGRNAGGVAGFDFDIAIHLGAGAYV
CGEESALIESLEGQRGTPRIRPPFPVQRGYLGRPTVVNNVETLVAVAHIARRGGAWWAGLGTPESTGTK
IHSVSGDCERPGLYEYPLGTPLAQILADCGARNAQAVQVGGPSGSCVPASGFHRAIAFEDLPSAGALMV
FDQTRDLFEVARHFARFFAHESCGLCTPCRVGTELVVRRLDKLAHERGAGRGSAFDIARLQELDALLHS
GTHCGLGVSACNPLRDTLAHFGQAYAQRSTAAQFQPGIDLDAELSSARRATGRQDRGAHLNTEHGA**

HoxU:

MTTFDFDGQPVPLQPGDTILQAAQRAGHEVPHLCWHEGVSASASCRLCTVVADGRPVPACATPAVAG
QRVECHTEALKTRRLHLLQMLFVEGNHFCPGCEKSGNCQLQHQAERAGMTDLHYEPLHPERPVDASH
PEVWFEPNRCILCQLCVRASDELDGKRVFAIGGHGIGAKLLIDSESGRLGDSQLSVEDRAAHICPVGALL
PKRVGFAQPYGQRTFDDAETRG**

HoxY:

MNTDSTPRKWRVATVSLAGCFGCHMSFLDIDERLFGLIEHITFDRSPLTDIKTVGPCDIGLIEGGLCNAEN
VEVLRAFRDQCRVLVAVGACAITGGLPALRNHLDVGEMMKAVYGEVPNDPELPLPLNRVRPIHEVVQI
DHALPGCPPPADAFWQLLQDLMAGREPKLHKGLIRYD

HoxH:

MTDAARPLETAADPQGLRRIVIDPVSRVEGHGKVTLLLDEQQRLQQVRLHIVEFRGFEQFIVGRPYWEV
PVMVQRLCGICPVSHHLAASKALDRVVGGWPVPEAADRIRRLMQYGQIVQSHALHFFHLSSPDLLFGF
DADVAQRNIVGVAMAHPEAARQGVMLRKFGQEVIRITSGKRVHGTGSVPGGMNRAVAREDRDTLRA
QLPEVLAWAEAAVELAQRLHTGLPPAYEHFGETPAAMMSLIGPGGAMELYDGALRLREADGRIAVDG
FEDQRYRELIDEAVKPWTYMKFPYRRALGPEAGWYRVGPLARLQNCDHIPTPRAEARRQAFVAAHGG
RPVHAVLATHWARMIELLHGVEVIARLLDDPVILGGPLQATGERQRSGVGIIEAPRGTLIHEYEVGDDD
LVKSCNLIVSTTHNNQAMNEAVRSVALQYLDGQTITEPLLNHLEVAIRAYDPCLSCATHALGQMPLSVT
LRGPDGEVLDHVLRSSTGETQRGATPHPMERAQ

***Tx*SH**

HoxF:

MQTTVTPDPAGPIPAVLARHGHRPHRLLQILHDVQDLYGWLPPEALTAVAHGLHLPRVAVESTAGFYS
FLYTRPVGQYRVLFSDNITDCLLGAPALMQRLCERLWLEPGKVSPDGLASVATTSCTGLCDQGPALLV
NGRAMPRVTPERIDAMAGLIQQRVPVADWPEDWFAIEDRIHRRDVLLRHVWRPGEVLQAARARGAQG
VLNEVARSGLRGRGGAGFPTGFKWRACAEAPGAQRVIVCNADEGEPGTFKDRVLLSTAFDLVVDGMC
VAALAVGARLGFLYLRAEYRYLLPRLQARLAERRAQGLLGAGCLGPGLDFDIDIHLGAGAYICGEESAL
IESLEGKPGKPRIRPPFPVTQGYLGRPTTVNNVETLATAALVALHGGDWLRAIGTESSSGTKVLSVSGDV
ARPGIYECPFGTPLAEVLHAAGAADVQAVTTAGAAGPCLSAGELHRRIAFEDVATGGSIMVFDRSRDLF
ELAHNVAHFFAHESCGFCTPCRVGTAVNARLLDKLAAHRGSPYDLEEMDRMHRLMQGASHCGLGNT
ATLALQDLARKFRPAFERRLASTTYEPAFDLDAALSEARRMTRRDDALAHLGSNRSAGLGQTP

HoxU:

MSTPSASPTFLLDGQPVPFTPGQTVMQAAYAAGRYIAHLCWHPDFAAHGSCKLCTVQANGRLATGCTL
AAAEGMEVRTQTPELEEKRRTLLQLLFVEGNHFCPACEKSGNCQLQATAYEAGMLTPHFDHFFPDRPL
DASHPDVLLDFNRCILCELCVRASRDVDGKHVFALSGRGTQSHLIVNSPTGRLADTDFAATDRAADICP
VGVILKKRVGFAVPIGQRRYDAQPASAVDVAPSSGGDA

HoxY:

MNPPVDTPMATPPRKLKIATVSLAGCFGCHMSFLDIDERLLPLLDLVEFDRSPLTDIKHCGPCDIGLIEGG
VCNAENVHVLREFRAQCRVLVALGACAVNGGLPAQRNHLAVGDVLQQVYITGHGLAPGSQIPNDPEL
PLPLNQVHPVHEVVRIDYFLPGCPPSGDAIWAFLNDLIAGREPRLGHGLLHYD

HoxH:

MTPAADLETAQVPREGLRRVAIDPVSRVEGHGKVTLLLDEQHRVHQVRLHIVEFRGFERFIQGRPYWE
VPVMVQRLCGICPVSHHLAASKALDAVVGARQLTPTAEALRRLMHYGQILQSHALHFFHLSSPDLLFGF
ESEVERRHLMGVAQAHPEIAKQGILLRKYGQEVIRLTSGKRVHGTASVPGGVNKALTGAERLALQQGI
GQVIDWSRQAVALVQRLHEQNPALYDTFGSFPSNFMGLVAPDGSLDLYDGALRATGADGQRLFDQFD
VRGYDRLLTEAVKPWTYMKFPYFTALGPEQGWYRVGPLARIQNADRLPTPLAEQARQQLLAYARARG
HAMLHATLAYHWARMIEMLHAAETIERLLHDDALEGTDLMTQGERPLAEVREGVGVIEAPRGTLIHH
YQVGDDDLVTMANLIVSTTHNNQAMNTAVREVAQRYLDGRELTEGLLNHIEVAIRAYDPCLSCATHA
LGKMPLEVVLLDADGTELDRRLRPGGALLKP**

Purity of additional purification of the novel SH

Due to the original low activity of the purified hydrogenases, CmSH, HpSH and TxSH were reproduced Cm- and HpSH while co-expressing their respective native HoxW, TxSH was purified anaerobically. To test for purity and changes in their subunit distribution, these three enzymes were analysed via SDS-PAGE. Unfortunately, The method chosen to produce TxSH anaerobically resulted low quantities and concentration, which did not allow an appropriate analysis via SDS gel. CmSH and HpSH are shown in Fig. S5 below.
In contrast to Fig 4 of the main text, there is a clear double band visible for HoxH, indicating a partial maturation by HoxW.

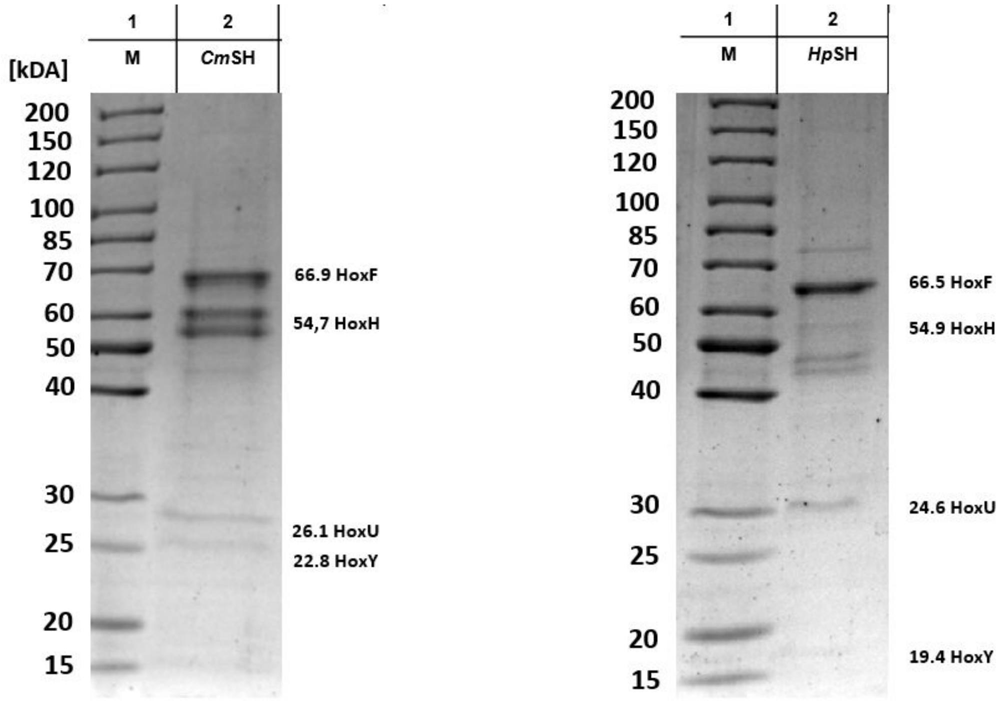

Figure S5. SDS-PAGE of the purified novel hydrogenases CmSH (left) and HpSH (right) after co-expression with respective native HoxW.
